# The Bacterial Communities of Little Cigars and Cigarillos Are Dynamic Over Time and Varying Storage Conditions

**DOI:** 10.3389/fmicb.2019.02371

**Published:** 2019-10-25

**Authors:** Eoghan M. Smyth, Suhana Chattopadhyay, Kelsey Babik, Molly Reid, Jessica Chopyk, Leena Malayil, Prachi Kulkarni, Lauren E. Hittle, Pamela I. Clark, Amy R. Sapkota, Emmanuel F. Mongodin

**Affiliations:** ^1^Maryland Institute for Applied Environmental Health, School of Public Health, University of Maryland, College Park, College Park, MD, United States; ^2^Institute for Genome Sciences, University of Maryland School of Medicine, Baltimore, MD, United States; ^3^Department of Behavioral and Community Health, School of Public Health, University of Maryland, College Park, College Park, MD, United States

**Keywords:** little cigars, cigarillos, tobacco components, bacterial microbiota, bacterial exposure, 16S rRNA gene sequencing analysis

## Abstract

Despite their potential importance with regard to tobacco-related health outcomes, as well as their hypothesized role in the production of tobacco-specific *N*-nitrosamines, bacterial constituents of tobacco products lack characterization. Specifically, to our knowledge, there has been no comprehensive characterization of the effects of storage conditions on the bacterial communities associated with little cigars and cigarillos. To address this knowledge gap, we characterized the bacterial community composition of the tobacco and wrapper components of the following four products: Swisher Sweets Original; Swisher Sweets, Sweet Cherry; Cheyenne Cigars Full Flavor 100’s; and Cheyenne Menthol Box. Each product was stored under three different conditions of temperature and relative humidity to mimic different user storage conditions: room (20°C 50% RH), refrigerator (5°C 18% RH) and pocket (25°C 30% RH). On days 0, 5, 9 and 14, subsamples were collected, the wrapper and tobacco were separated, and their total DNA was extracted separately and purified. Resulting DNA was then used in PCR assays targeting the V3 V4 region of the bacterial 16S rRNA gene, followed by sequencing using Illumina HiSeq 300bp PE. Resulting sequences were processed using the Quantitative Insights Into Microbial Ecology (QIIME) software package, followed by analyses in R using the Phyloseq and Vegan packages. A single bacterial phylum, Firmicutes, dominated in the wrapper subsamples whereas the tobacco subsamples were dominated by Proteobacteria. Cheyenne Menthol Box (CMB) samples were characterized by significant differential abundances for 23 bacterial operational taxonomic units (OTUs) in tobacco subsamples and 27 OTUs in the wrapper subsamples between day 0 and day 14 under all conditions. OTUs from the genera *Acinetobacter* and *Bacillus* significantly increased in the CMB tobacco subsamples, and OTUs from *Bacillus*, *Streptococcus*, *Lactobacillus*, and *Enterococcus* significantly increased in the CMB wrapper subsamples over time. These initial results suggest that the bacterial communities of little cigars and cigarillos are dynamic over time and varying storage conditions.

## Introduction

Little cigars and cigarillos are popular tobacco products commonly smoked in North America and throughout the world (Ayo-Yusuf and Burns, 2012). In the U.S., it is estimated that 3.6% of adults 18 years and older have smoked little cigars, and these rates of usage have increased significantly since 2000 (USDHHS, 2014). Their use is also more prevalent among African Americans, lower socio-economic populations and current users of other tobacco products (Nyman et al., 2016). Little cigars resemble cigarettes but are defined as any tobacco roll wrapped in leaf tobacco, reconstituted tobacco, or any substance (usually paper) containing tobacco for which 1000 units weigh less than three pounds (Conolly, 1998; Messer et al., 2015). Cigarillos are more like traditional cigars, containing about 3 g of tobacco wrapped in tobacco leaves or brown paper mixed with tobacco leaves. Cigarillos are thinner and smaller in diameter than a traditional cigar but longer than a normal cigarette, and, unlike traditional cigars, cigarillos are smoked with a deeper inhalation. The use of little cigars and cigarillos varies widely depending on sex, age, ethnic origin, and socio-economic status but their use has steadily increased among young adolescents (Cullen et al., 2011) because they are cheaper than cigarettes and they are taxed at a lower rate. A common misconception is that little cigars and cigarillos are generally thought to be less toxic than cigarettes. However, because little cigars contain more tobacco than cigarettes, are smoked for longer lengths of time, and contain higher levels of carcinogens, they are associated with the same adverse health effects as cigarettes. These include addiction to nicotine, oral lesions, oral and pancreatic cancer, chronic obstructive pulmonary disease (COPD), cardiovascular disease, and lung cancer (World Health Organization [WHO], 2006; O’Connor, 2012). Recent studies have suggested that they may actually be more harmful to human health than cigarettes (Ghosh et al., 2017).

The chemical constituents of little cigar and cigarillo mainstream smoke have not been studied as widely as that of cigarettes, but it is estimated that it contains as many chemicals as cigarette mainstream smoke (Talhout et al., 2011). A recent study demonstrated that little cigar mainstream smoke contains over 4,000 distinct chemicals (Klupinski et al., 2016). Similar to cigarettes, many of the adverse health outcomes of smoking little cigars can be linked to chemical carcinogens present in little cigar tobacco, such as tobacco-specific nitrosamines (TSNAs), nitrosamino acids and polycyclic aromatic hydrocarbons (Stepanov et al., 2008; Talhout et al., 2011; Klupinski et al., 2016). TSNAs have been shown to be among the most important tobacco-associated carcinogens due to their toxicity and high abundance in tobacco products (Stepanov et al., 2012). TSNAs, such as 4-(methylnitrosamino)-1-(3-pyridyl)-1-butanone (NNK) and N′-nitrosonornicotine (NNN), are typically formed through nitrosation reactions of tobacco alkaloids (such as nicotine and nornicotine) (Wei et al., 2014) during curing and storage of tobacco leaves. It should be noted that levels of available nitrite in the tobacco are primarily influenced by nitrite-reducing bacteria, which are part of the normal consortium of bacteria (i.e., the microbiota) associated with tobacco products (Fisher et al., 2012). As such, TSNA production is critically influenced by the bacterial communities associated with the tobacco.

Several recent studies using next-generation sequencing have characterized the bacterial communities in a variety of tobacco products, including smokeless tobacco (Han et al., 2016; Tyx et al., 2016; Smyth et al., 2017), cigarettes (Chopyk et al., 2017a, b) and little cigars (Chattopadhyay et al., 2019). Our group recently showed that cigarette tobacco was dominated by bacteria from the genera *Pseudomonas*, *Pantoea*, and *Bacillus*, many of which contain potentially pathogenic species – such as *Pseudomonas putida*—that cigarette smokers may be exposed to over time (Chopyk et al., 2017a, b). Smokeless tobacco has been shown to be dominated by bacteria from the *Firmicutes*, *Proteobacteria*, *Actinobacteria*, and *Bacteroidetes* phyla (Han et al., 2016; Tyx et al., 2016; Smyth et al., 2017). In an initial cross-sectional study focused on identifying the microbiota present in little cigars and cigarillos (Chattopadhyay et al., 2019), we have shown that little cigars are composite products in regards to their microbiota, with the tobacco being dominated by bacteria from the genera *Pantoea, Pseudomonas* and *Staphylococcus*, and the little cigar wrapper being dominated by bacteria from the *Lactobacillus* and *Bacillus* genera.

In cigarette products, we have previously shown that different storage conditions can significantly influence the bacterial composition of cigarettes over time (Chopyk et al., 2017b), potentially increasing exposures to certain bacterial species among cigarette smokers. Because such temporal characterizations are currently lacking for little cigar products, the aim of this study was to characterize the bacterial microbiota longitudinally across different little cigar brands and under varying storage conditions using a next-generation sequencing approach.

## Materials and Methods

### Sample Collection and Treatment

In the Spring of 2015, four different little cigar products were purchased online and shipped to College Park, Maryland; Cheyenne Full Flavor, Cheyenne Menthol Box (Cheyenne International, LLC, Grover, NC, United States); Swisher Sweets Sweet Cherry and Swisher Sweets Original (Swisher International, Inc., Jacksonville, FL, United States) (Table 1). Little cigars were subjected to three different experimental conditions over a 14-day period to mimic user storage: pocket (25°C, 30% relative humidity), room (20°C, 50% relative humidity) and refrigerator (5°C, 18% relative humidity). Three lots of each little cigar product (except for the Swisher Sweets Original product, for which only two lots were characterized) were sampled with duplicate samples for both the wrapper and the tobacco at each condition and time point (days 0, 5, 9, and 14).

**TABLE 1:** Little cigar brands and tobacco components tested in this study.

**Product brand**		**Component**		**Manufacturer**
Cheyenne Full Flavor	(CFF)	Tobacco	(T)	Cheyenne International, LLC
Cheyenne Full Flavor	(CFF)	Wrapper	(W)	Cheyenne International, LLC
Cheyenne Menthol Box	(CMB)	Tobacco	(T)	Cheyenne International, LLC
Cheyenne Menthol Box	(CMB)	Wrapper	(W)	Cheyenne International, LLC
Swisher Sweets Sweet Cherry	(SSC)	Tobacco	(T)	Swisher International, Inc.
Swisher Sweets Sweet Cherry	(SSC)	Wrapper	(W)	Swisher International, Inc.
Swisher Sweets Original	(SSO)	Tobacco	(T)	Swisher International, Inc.

### DNA Extraction

DNA extraction was performed on little cigar products from freshly opened packages using procedures published previously (Chopyk et al., 2017a, b). Each little cigar was dissected under sterile conditions, to analyze separately its two main components: tobacco and wrapper. The wrapper was separated from the internal tobacco and 0.2 g of each was weighed out separately into Lysing Matrix B tubes (MP Biomedicals, Solon, OH, United States). Enzymatic lysis was then initiated by adding the following to the tubes containing either little cigar tobacco or wrapper, and lysing matrix: 1 ml of ice cold 1X molecular grade PBS buffer (Gibco by Life Technologies, Grand Island, NY, United States), 5 μl lysozyme from chicken egg white (10 mg/ml, Sigma-Aldrich, St. Louis, MO, United States), 5 μl lysostaphin (5 mg/ml, Sigma-Aldrich, St. Louis, MO, United States) and 15 μl of mutanolysin (1 mg/ml, Sigma-Aldrich, St. Louis, MO, United States). Tubes were then incubated at 37°C for 30 min, after which a second enzymatic cocktail was added to each tube, composed of 10 μl Proteinase K (20 mg/ml, Invitrogen by Life Technologies, Grand Island, NY, United States) and 50 μl of SDS (10% w/v, Bio-Rad, Hercules, CA, United States). Following incubation at 55°C for 45 min, the samples were mechanically lysed using a FastPrep Instrument FP-24 (MP Biomedicals, Santa Ana, CA, United States) at 6.0 m/s for 40 s. The resulting lysate was centrifuged for 3 min at 10,000 rcf and the resulting supernatant was purified for DNA using the QIAmp DSP DNA mini kit 50, v2 (Qiagen, Valencia, CA, United States), according to the manufacturer’s protocol. Duplicate DNA extractions were completed on each sample and negative extraction controls were included to ensure that no exogenous DNA contaminated the samples during extraction. DNA quality control/quality assurance was performed using spectrophotometric measurements on a NanoDrop^TM^ (Thermo Fisher Scientific, Waltham, MA, United States).

### 16S rRNA Gene PCR Amplification and Sequencing

The V3–V4 hypervariable region of the 16S rRNA gene was PCR-amplified and sequenced using a dual-indexing strategy for multiplexed sequencing developed at the Institute for Genome Sciences (Fadrosh et al., 2014) and used in our previous studies aimed at characterizing the bacterial communities in various tobacco products (Chopyk et al., 2017a, b). Briefly, PCR reactions were set-up in 96-well microtiter plates using the 319F (ACTCCTACGGGAGGCAGCAG) and 806R (GGACTACHVGGGTWTCTAAT) universal primers, each of which also included a linker sequence required for Illumina sequencing, and a 12-bp heterogeneity-spacer index sequence aimed at minimizing biases associated with low-diversity amplicons sequencing (Caporaso et al., 2012; Fadrosh et al., 2014). PCR amplifications were performed using Phusion High-Fidelity DNA polymerase (Thermo Fisher Scientific, Waltham, MA, United States) and 2 ng of template DNA in a total reaction volume of 25 μl. Because of the potential presence of PCR inhibitors in the DNA solution, an additional 0.375 μl of bovine serum albumin (BSA) (20 mg/ml, Sigma) was added to the PCR reactions. Reactions were run in a DNA Engine Tetrad 2 thermo cycler (Bio-Rad, United States) using the following cycling parameters: 30 s at 98°C, followed by 30 cycles of 10 s at 98°C, 15 s at 66°C, and 15 s at 72°C, with a final step of 10 min at 72°C. Negative controls using molecular grade water and no DNA template were performed for each primer pair. The presence of amplicons was confirmed using gel electrophoresis, after which the SequalPrep Normalization Plate kit (Invitrogen, Inc., Carlsbad, CA, United States) was used for clean-up and normalization (25 ng of 16S PCR amplicons from each sample were included), before pooling and 16S rRNA sequencing using the Illumina HiSeq 2500 300bp paired-ends (Illumina, San Diego, CA, United States). Despite multiple attempts, no 16S rRNA gene amplicons could be generated for the Swisher Sweets Original wrapper DNA samples, most likely due to very low amounts of bacterial target DNA, and therefore these samples were removed from the analysis.

### Sequence Quality Filtering and Analysis of 16S rRNA Reads

16S rRNA reads were initially screened for low quality bases and short read lengths. Paired-end read pairs were then assembled using PANDAseq (Masella et al., 2012) and the resulting consensus sequences were de-multiplexed, trimmed of artificial barcodes and primers, and assessed for chimeras using UCHIME in *de novo* mode implemented in Quantitative Insights Into Microbial Ecology (QIIME; release v. 1.9.1) (Caporaso et al., 2010). Quality trimmed sequences were then clustered *de novo* into operational taxonomic units (OTUs) using VSEARCH (Rognes et al., 2016) with a minimum confidence threshold of 0.97, and taxonomy assigned using the Silva database (Quast et al., 2013) in QIIME (Caporaso et al., 2010). The resulting OTU table, OTU reference sequences and phylogenetic tree files were imported to the R Statistical computing software (v. 3.4.3) (R Developmental Core Team, 2010) using the Phyloseq R package (v. 1.22.3) (McMurdie and Holmes, 2013). All sequences taxonomically assigned to the Phylum *Cyanobacteria*, and likely tobacco chloroplast sequences, were removed from further downstream analysis. Tobacco microbiota alpha-diversity was characterized using the Observed richness metric calculated through the Phyloseq R package (McMurdie and Holmes, 2013). Significant differences in alpha-diversity were tested using ANOVA with Tukey’s HSD (Honestly Significant Difference) *post hoc* test. For beta-diversity, data was normalized to account for uneven sampling depth with metagenomeSeq’s (v. 1.20.1) cumulative sum scaling (CSS) (Paulson et al., 2013). Differences in beta-diversity were then determined though principal coordinates analysis (PCoA) and t-distributed stochastic neighbor embedding (t-SNE) (van der Maaten and Hinton, 2008) plots of Bray–Curtis dissimilarity calculated using the Vegan v. 2.4.5 (Oksanen et al., 2007), tsnemicrobiota (v. 0.1.0) and Phyloseq (McMurdie and Holmes, 2013) R packages, and tested for significance with ANOSIM (999 permutations) (Clarke and Warwick, 2001). Determination of statistically significant (*p-*value cutoff of 0.05) differences in the average axis-1 PCoA coordinate for each brand, component and condition over time was tested using the Tukey HSD test in R.

Determination of statistically significant (*p* value cutoff of 0.05) differences in bacterial OTU composition between day 0 and day 14 for all brands and components of tobacco was performed using the R package DESeq2 (v. 1.18.1) at alpha = 0.05 (Love et al., 2014) on OTUs present at greater than 0.1% abundance. Data were visualized with RStudio (v. 1.1.383) and R packages ggplot2 (v. 2.2.1) (Wickham, 2009), Phyloseq (McMurdie and Holmes, 2013), and UpSetR (v. 1.3.3) to visualize the interactions of the core microbiome (Lex et al., 2014). Shared core OTUs were defined as the OTUs present across all the tobacco brands, components, time points, and storage conditions. Biomarker OTUs were defined as the OTUs present in 100% of the samples from combination of tobacco brands, components, time points, and storage condition samples compared and absent from all others.

#### Availability of Data

Data concerning the samples included in this study are deposited in the NCBI BioProject database under BioProject accession number PRJNA473598.

## Results

### 16S rRNA Gene Sequencing Dataset

Bacterial community profiling using 16S rRNA gene sequencing was performed on a final dataset comprising a total of 456 little cigar samples, which included 4 little cigar products (Table 1), 3 lots per product, 2 components per little cigar (tobacco and wrapper) per condition and time point, with the exception of Swisher Sweets Sweet Cherry (only 2 lots tested) and Swisher Sweets Original (tobacco component only). For Swisher Sweets Original wrappers, 16S rRNA gene PCR amplification consistently failed (Supplementary Table S1) despite multiple attempts, most likely due to very low bacterial burden. A total of 24,141,006 16S rRNA gene sequences were obtained, representing 6,507 unique OTUs at a 97% similarity cut-off across all samples. On average, 52,941 sequences (min 17 – 108,776 max) were obtained per sample, which could be clustered to 660 OTUs (min 16 – 1,372 max) on average per sample. After OTU clustering and taxonomic assignments, OTUs assigned to the phylum *Cyanobacteria* (137 OTUs associated to 3,513,576 sequences) were removed from further downstream analysis, as these mostly represent sequences amplified from tobacco chloroplast DNA (∼99.91% of sequences taxonomically classified to the class *Chloroplasts*). To ensure samples included in the final dataset were sequenced near saturation, the Good’s coverage index was calculated for each sample, and samples with Good’s ≤0.9 (19 samples) were removed from further downstream analyses (Supplementary Table S1). The resulting final dataset was further cleaned up by filtering out OTUs with less than 10 reads in total across all samples, which are likely to be spurious OTUs (1,916 OTUs associated to 9,277 sequences).

### Baseline Cross-Sectional Characterization of the Bacterial Microbiota in Little Cigars

**FIGURE 1:**
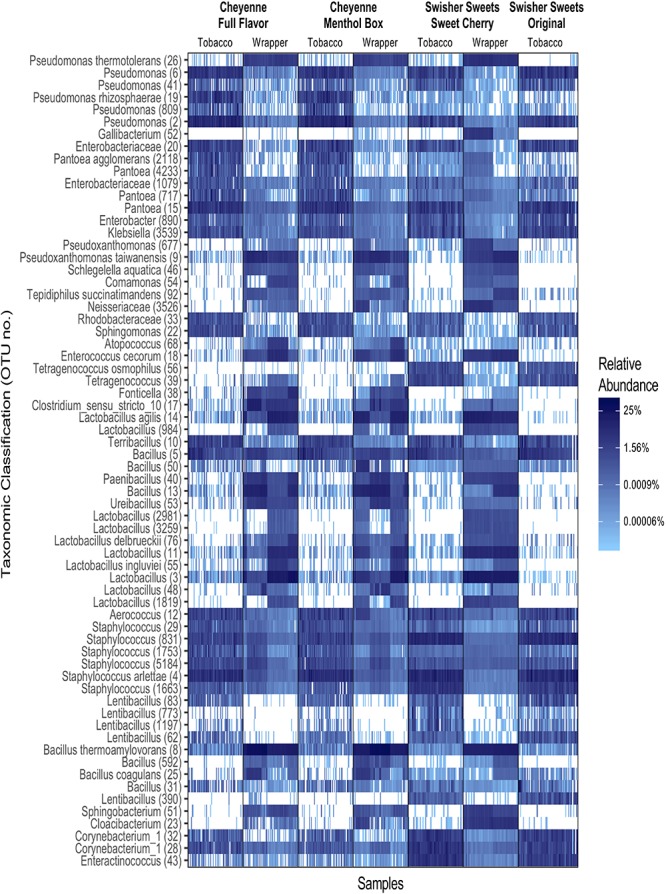
Heatmap displaying the normalized relative abundance of the top 20 operational taxonomic units (OTUs) in each tobacco and wrapper components across the little cigar brands tested. OTU taxonomic assignments were performed at the species level whenever possible, or a higher taxonomic level whenever species-level assignment was not possible. Numbers between parentheses represent OTU numbers.

Detailed cross-sectional analysis of the baseline (day 0) microbiota associated with different brands, lots, and components of little cigar products was performed in our previous cross-sectional study (Chattopadhyay et al., 2019). Briefly, the heatmap displayed in Figure 1 summarizes the differences in bacterial community composition between the different brands of little cigars, and between tobacco and wrapper. For clarity, this heatmap was constructed after selecting only the top 20 most abundant OTUs from each little cigar brand and component, yielding a combined dataset of 66 OTUs in total and representing 67.0% of all the 16S rRNA sequences in our dataset.

The heatmap in Figure 1 highlights the differences in bacterial community composition between the little cigar tobacco and wrapper. Among these differences, OTUs from the *Enterobacteriaceae* class, and from the genera *Pantoea, Pseudomonas* and *Staphylococcus*, were more abundant in little cigar tobacco compared to the little cigar wrapper (Figure 1). For each little cigar brand, the average relative abundance of *Enterobacteriaceae* OTUs ranged between 11.5–20.6% (mean 3.62 ± 3.56 SD) in tobacco and 0.6–1.7% (mean 0.10 ± 0.35 SD) in the wrapper. The average relative abundance of *Pantoea* OTUs ranged between 4.2–11.9% (mean 7.69 ± 7.94 SD) in tobacco and 0.3–0.4% in wrapper. *Pseudomonas* OTUs ranged between 1.7–4.1% (mean 12.32 ± 8.82 SD) in tobacco and 11.5–20.6% (mean 2.55 ± 1.18 SD) in wrapper. The average relative abundance of *Staphylococcus* OTUs ranged between 11.5–20.6% (mean 14.87 ± 9.19 SD) in tobacco and 0.6–1.7% (mean 1.28 ± 1.56 SD) in wrapper.

In contrast, OTUs from the genera *Lactobacillus* and *Bacillus* were more abundant in the little cigar wrapper than in the tobacco, across all brands, conditions, and storage days (Figure 1). The relative abundance of *Bacillus* ranged between 3.5–6.3% (mean 3.51 ± 6.03 SD) in tobacco and 16.1–30.9% (mean 28.97 ± 16.33 SD) in wrapper. The average relative abundance of *Lactobacillus* OTUs ranged between 0.0–0.4% (mean 0.17 ± 0.48 SD) in tobacco and 5.9–18.1% (mean 27.38 ± 16.29 SD) in wrapper. These initial analyses clearly demonstrated that little cigar products are composite products in regards to their microbiota, with tobacco and wrapper components of little cigars having significantly distinct bacterial signatures.

### Characterization of the Little Cigar Microbiota Over Time and Storage Conditions

**FIGURE 2:**
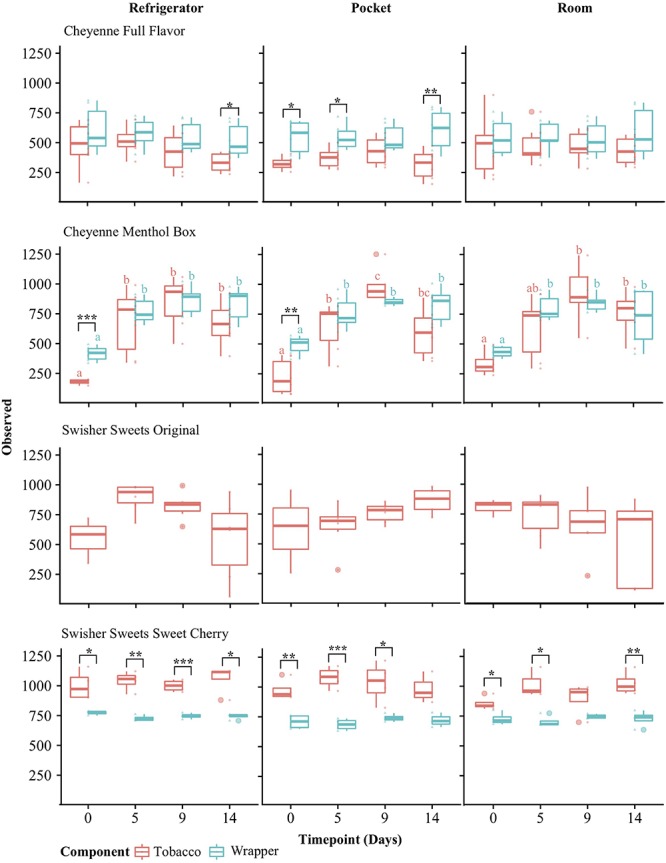
Alpha-diversity analysis of little cigar brands and components over time and under different storage conditions. Alpha-diversity was measured for tobacco (red) and wrapper (blue) components using the Observed richness metric and compared using ANOVA with Tukey’s HSD (honestly significant difference) *post hoc* test. Significance levels: ^∗^*p* < 0.05; ^∗∗^*p* < 0.01; ^∗∗∗^*p* < 0.001. The letters over the boxes denote statistical significance assessed by Tukey’s HSD test comparing time points for each tobacco component: time points with different letters are significantly different (*p* < 0.05) in their Observed richness, whereas time points with the same letter indicate no significant differences.

Alpha-diversity metrics have been used in ecology studies to characterize within-sample biodiversity. One such measure, the observed organismal richness, is defined in microbial ecology as the number of species within a sample. The observed alpha-diversity varied for each brand and tobacco component over time (Days 0, 5, 9, and 14) and storage conditions (refrigerator, room, and pocket) (Figure 2). Observed alpha diversity for Swisher Sweets, Sweet Cherry tobacco was significantly higher than that of wrappers (*p* < 0.05) for all conditions and time points except day 14 under room and day 9 under pocket conditions (Figure 2). However, observed alpha diversity was consistently higher for Cheyenne Full Flavor wrappers for each condition and time point, and was also significantly different on day 14 under refrigerator conditions and days 0, 5, 9 under pocket conditions (Figure 2). Observed alpha diversity varied between Cheyenne Menthol Box tobacco and wrappers for each condition and time point with observed diversity for wrappers being significantly different than that of tobacco at day 0 under refrigerator and pocket conditions (*p* > 0.01). Changes in alpha diversity over time, were mainly seen in Cheyenne Menthol Box for both tobacco and wrappers (Figure 2). Significant differences were found between day 0 and days 5, 9, and 14 across all conditions and both tobacco and wrappers (*p* < 0.05).

Beta diversity analyses were then used to perform between-sample comparisons and characterize time-dependent shifts of the overall structure of the little cigar microbiotas, using the Bray–Curtis dissimilarity measure plotted using PCoA. Little cigar component (i.e. tobacco or wrapper) explained the greatest variance in bacterial microbiota between samples (ANOSIM: *R* = 0.1118, *p* = 0.001) (Supplementary Figure S2). We also used t-stochastic neighbor embedding (t-SNE) analysis to look at the similarity between samples. t-SNE is a novel non-linear, non-parametric dimensionality reduction technique used to visualizes high-dimensional data (van der Maaten and Hinton, 2008), and that has been shown to perform better than currently used approaches to reveal data-inherent cluster structures in complex datasets. Clustering with t-SNE confirmed that that tobacco and wrapper samples have significantly different microbiota, but also showed smaller groups of wrapper samples clustering mainly by lot (Supplementary Figure S2), highlighting lot-to-lot variation in microbiota that was not apparent using PCoA. The next greatest variation was seen between little cigar lots, particularly noticeable in the little cigar wrappers (ANOSIM: *R* = 0.1118, *p* = 0.001) (Supplementary Figure S3).

**FIGURE 3:**
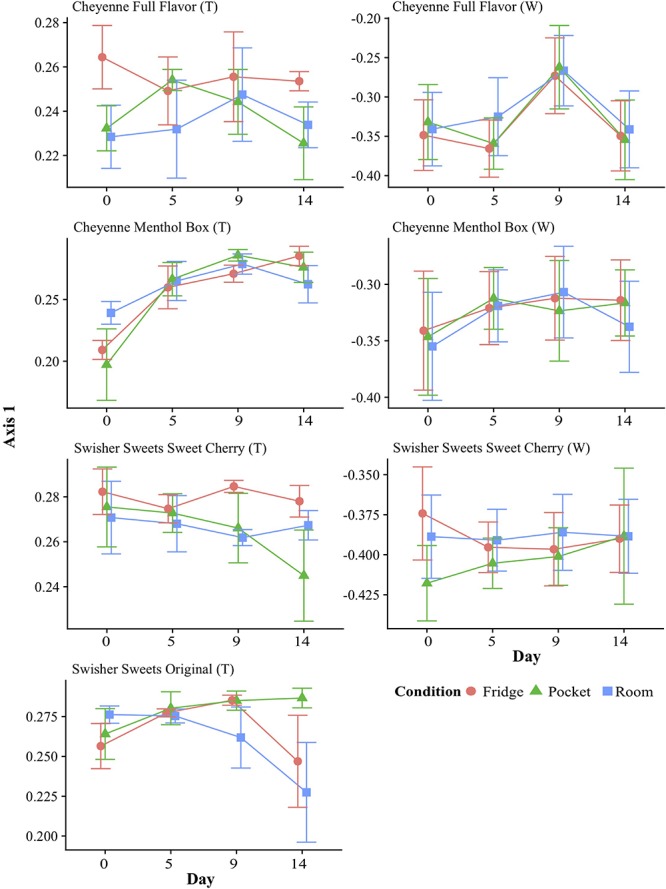
Changes in beta-diversity over time and under different storage conditions. Beta-diversity was calculated using principal coordinates analysis (PCoA) of Bray–Curtis dissimilarity, and averages of PCoA Axis 1 values were plotted over time for each brand and component (T: tobacco; W: wrapper) of little cigars stored under different conditions. PCoA axis 1 was chosen as it captures most of the variation in dissimilarity in the dataset (40.7% of variation explained by Axis 1 – Supplementary Figure S2A) and with the highest eigenvalues (Supplementary Figure S1). The microbiota from the tobacco component of Cheyenne Menthol Box little cigars fluctuated significantly (*p* < 0.05 Tukey HSD test) between day 0 and days 5, 9, and 14 for refrigerator and day 0 and day 9 for pocket conditions.

To display changes in bacterial community structure over time, the mean coordinate of axis 1 of the PCoA (which showed the greatest variance (Supplementary Figure S1) was plotted over time for each tobacco component and condition. In general, these time-dependent fluctuations in overall bacterial community structure were similar for each condition (Figure 3). However, the greatest shifts over time could be seen in Cheyenne Menthol Box tobacco. Significance over time for each little cigar brand component and condition was tested using Tukey HSD. This analyses showed that the Cheyenne Menthol Box tobacco microbiota fluctuated significantly over the 14 days of storage between day 0 and days 5, 9, and 14 for refrigerator and day 0 and days 9 for pocket conditions (*p* < 0.05).

### Differential Abundance of OTUs Over Time Under Different Storage Conditions

**FIGURE 4:**
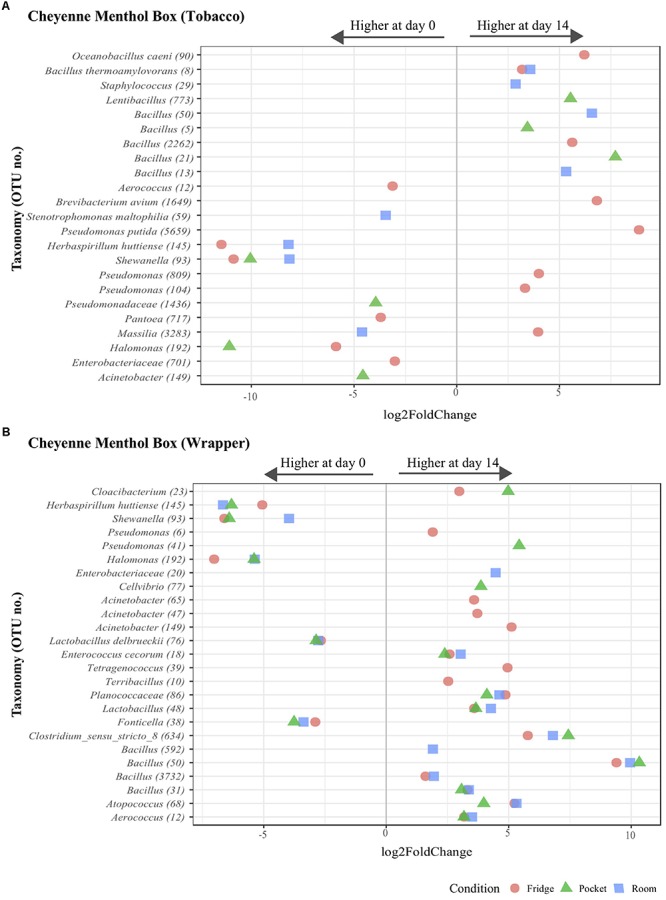
Operational taxonomic unit differential abundance analysis for the microbiota of Cheyenne Menthol Box tobacco and wrapper components. Differential abundance plots showing the significant (assessed using DESeq2 at level *p* < 0.05) log2 fold changes of OTUs under all storage conditions between day 0 to day 14 for both Cheyenne Menthol Box tobacco **(A)** and wrapper component **(B)**. Colors indicate the storage conditions. Numbers between parenthesis for the taxa shown on the Y axis represent OTU.

Differential abundance analyses were performed to more precisely characterize the differences in microbiota composition and shifts over time under different storage conditions. For these analyses, we considered OTUs with greater than 0.1% relative abundance (135 OTUs in total, accounting for 78.6% of all sequences) and compared days 0 to 14 for all little cigar brands, components and storage conditions. Significant differential abundance between days 0 and 14 was only found for the Cheyenne Menthol Box brand, for both components and all conditions (Figure 4). Nineteen OTUs were significantly differentially abundant in Cheyenne Menthol Box tobacco and 43 were significantly differentially abundant in Cheyenne Menthol Box wrapper, 10 OTUs were significantly differentially abundant in both (Figure 4).

Most of the significantly differentially abundant OTUs were identified in the “refrigerator” condition, 6 OTUs were significantly higher on day 0; *Aerococcus* (12), *Herbaspirillum huttiense* (145), *Shewanella* (93), *Pantoea* (717), *Halomonas* (192) and *Enterobacteriaceace* (701) (Figure 3). Eight OTUs were significantly higher on day 14 under “refrigerator” conditions in Cheyenne Menthol Box tobacco; *Oceanobacillus caeni* (90), *Bacillus thermoamylovorans* (8), *Bacillus* (2262), *Brevibacterium avium* (1649), *Pseudomonas putida* (5629), *Pseudomonas* (809 and 104), and *Massilia* (3283) (Figure 4). One OTU was significantly higher on day 0 for all conditions; *Shewanella* (93) in Cheyenne Menthol Box tobacco (Figure 3).

Five OTUs were significantly higher on day 0 for all conditions; *Herbaspirillum huttiense* (145), *Shewanella* (93), *Halomonas* (19), *Lactobacillus delbrueckii* (78), and *Fonticella* (38) (Figure 4) and seven OTUs were significantly higher on day 14 for all conditions; *Aerococcus* (12), *Atopococcus* (68), *Bacillus* (50), *Clostridium* (634), *Lactobacillus* (48), *Planococcaceae* (86), and *Enterococcus cecorum* (18) in Cheyenne Menthol Box wrapper (Figure 4). The average relative abundance of all the significantly differentially abundant OTUs were plotted over time showing the changes over time, at all 4 time points (Supplementary Figures S4, S5).

### Shared Microbiota of Little Cigar Brands and Components Over Time and Storage Conditions

**FIGURE 5:**
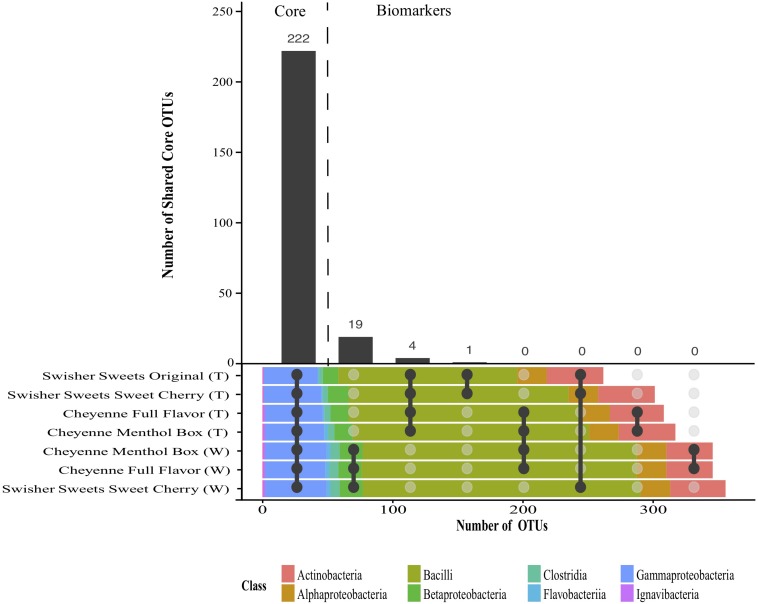
Interaction plot showing shared core and biomarker OTUs identified across the different little cigar brands and tobacco components. Shared core OTUs were identified as the OTUs present in all the tobacco brands, components, time points, and storage conditions. Biomarker OTUs were defined as the OTUs present in 100% of the samples from combination of tobacco brands, components, time points, and storage condition samples compared and absent from all others.

Despite the significant differences in microbiota composition across little cigar components, brands and storage conditions, shared core microbiotas could be identified for different combinations of little cigar brands and components (Figure 5). Shared microbiota was defined as the OTUs present in all the samples compared for all storage conditions and sampling days. Overall, a total of 222 OTUs were present in every little cigar brand and component samples at every time point and under every storage condition, and were considered as core bacterial OTUs for all little cigars brands and components. In addition, we identified 19 OTUs that were shared between all the little cigar wrapper samples but absent in little cigar tobacco samples, as well as 4 OTUs that were shared between all the little cigar tobacco samples and absent in the wrapper samples. These OTUs are considered as potential biomarkers for little cigar wrapper and tobacco, respectively. Of the 19 OTUs that were predicted as biomarkers for little cigar wrappers, 3 were taxonomically classified to the order *Bacillales*, 1 to the family *Enterococcaceae*, 2 to the order *Lactobacillales*, 12 to the genus *Lactobacillus* and 1 to the genus *Sporomusa*. These distinct biomarker OTUs for the little cigar wrappers had low abundance (<0.04% relative abundance on average) and were absent in all the other little cigar tobacco samples (Supplementary Figure S6). Of the 4 OTUs that were core in all little cigar tobacco, 1 OTU was taxonomically classified to the family *Bacillaceae*, 1 to the genus Bacillus, 1 to the family *Corynebacteriaceae* and 1 to the genus *Staphylococcus*. Bacterial biomarkers for manufacturers could only be identified for Swisher Sweets manufactured little cigars: 1 OTU, taxonomically classified to the genus *Tetragenoccus*, was present in the tobacco component of all Swisher Sweets manufactured little cigar samples (Figure 5). Bacteria from the *Tetragenoccus* genus are halophilic lactic acid bacteria that have been identified in various fermentation processes, and that have also been isolated from smokeless tobacco products (Han et al., 2016). *Tetragenoccus* bacteria also had very low relative abundance, but were absent in all the other little cigar brands and component samples (Supplementary Figure S6).

## Discussion

The present study sought to generate a comprehensive longitudinal characterization of the bacterial microbiota present in little cigar products under different storage conditions, using a cultivation-independent next-generation sequencing approach. In a previous study, where the microbiotas of little cigar tobacco and wrapper components were characterized cross-sectionally at baseline for different brands and lots (Chattopadhyay et al., 2019), we showed that little cigar products have a composite microbiota, where tobacco and wrapper components harbor significantly different bacterial microbiotas. The little cigar tobacco and wrapper components were shown to be significantly different in their alpha- and beta- diversity, as well as in their bacterial microbiota composition, for all little cigar brands tested. In this study, we further characterized the microbiota associated with the tobacco and wrapper of little cigars, focusing on time-dependent shifts occurring under different storage conditions. We confirmed the findings from our previous study and showed that the differences seen at baseline between wrapper and tobacco persisted over time under differing storage conditions. In addition, several bacterial OTUs were found to be significantly differentially abundant after 14 days for both tobacco and wrapper components of Cheyenne Menthol Box little cigars, suggesting that the little cigar microbiota is dynamic and can be influenced by environmental factors, such as temperature, humidity, and storage time.

Analysis of core bacterial communities, defined as the bacterial OTUs found in all brands, lots and timepoints, showed that the little cigar tobacco was characterized by a core microbiota composed of 222 bacterial OTUs, compared to only 19 bacterial OTUs for the little cigar wrapper core microbiota. Despite the variability seen in tobacco and wrapper microbiotas as influenced by brands, storage conditions and duration, these results suggest that each component (i.e., tobacco or wrapper) of little cigar products has a unique bacterial signature. Detailed analyses showed that OTUs from the class *Enterobacteriaceae* and the genera *Pantoea, Pseudomonas*, and *Staphylococcus* were more abundant in the little cigar tobacco compared to the little cigar wrapper. In addition, OTUs from the genera *Lactobacillus* and *Bacillus* are more abundant in the little cigar wrappers compared to little cigar tobacco, across all brands, conditions, and days.

Previous studies, have characterized the bacterial communities in a variety of tobacco products using next generation sequencing, including smokeless tobacco (Han et al., 2016; Tyx et al., 2016; Smyth et al., 2017), and cigarettes (Chopyk et al., 2017a, b). These studies have shown that different tobacco products also harbor a rich and diverse bacterial microbiota, with each product characterized by its own unique bacterial profile. Bacteria from the genera *Pseudomonas*, *Pantoea*, and *Bacillus*, were shown to dominate the cigarette tobacco (Chopyk et al., 2017a, b), and smokeless tobacco was shown to be dominated by bacteria from the phyla *Firmicutes*, *Proteobacteria*, *Actinobacteria*, and *Bacteroidetes* (Han et al., 2016; Tyx et al., 2016; Smyth et al., 2017), with significant differences in bacterial composition and diversity across smokeless tobacco product brands and types (Smyth et al., 2017). In two of these studies (Han et al., 2016; Smyth et al., 2017), bacteria were also cultured from smokeless tobacco products, indicating that at least a proportion of the bacteria characterized by culture-independent sequencing strategies are viable bacteria residing in smokeless tobacco products. Similar studies combining approaches that can distinguish the active and viable bacteria from dormant/dead cells in the tobacco microbiota would be of interest to further characterize the microbiota associated with little cigars, and more precisely assess the bacterial communities that users may be chronically exposed to when smoking little cigar products.

Although longitudinal shifts in bacterial relative abundances were seen for different storage conditions and little cigar products, differential abundance of bacterial OTUs between days 0 and 14 was only significant for Cheyenne Menthol Box tobacco and wrapper components, for all storage conditions. In total, 52 OTUs had differential abundance between days 0 and 14. Five OTUs from the genus *Bacillus* had a higher differential abundance at day 14 in the tobacco component and 4 OTUs in the wrapper component. These temporal shifts indicate that a portion of the bacterial microbiota is potentially dynamic over time depending on storage conditions. We previously showed that storage conditions can significantly affect bacterial relative abundance of the tobacco microbiota in various brands of commercially-available cigarettes (Chopyk et al., 2017b). In contrast with cigarette products, we only saw significant shifts for one little cigar brand – Cheyenne Menthol Box – in this study. This observation may be due to different types of tobacco and/or curing methods used in little cigars and cigarette manufacturing processes, affecting differently the resilience over time of the tobacco-associated microbiotas in each product type. In general, cigarettes contain a blend of flue-cured, air-cured tobacco, Maryland tobacco and reconstituted tobacco, while little cigars contain a blend of air-cured and fermented tobacco (Hoffmann and Hoffmann, 1998). It is also likely that the mentholation process that Cheyenne Menthol Box little cigars are subjected to may affect the bacterial microbiota over time, as menthol has been shown to act as a mild anti-microbial agent (İşcan et al., 2002).

Even though several OTUs showed longitudinal fluctuations in their relative abundance, a little cigar core microbiota, defined as the bacteria present in all brands, storage times and conditions, of 222 bacterial OTUs could be identified. We could also identify 19 biomarker OTUs unique to little cigar wrappers, as well as 4 biomarker OTUs for little cigar tobacco samples. Chopyk et al. (2017b) showed that cigarettes had a core microbiome over several different brands but demonstrated that only 11 OTUs were conserved across every cigarette sample. A comparison of the bacterial microbiota of all tobacco types; smokeless, cigarette, and little cigars would be of interest to see if there are common bacterial microbiota for all tobacco products and if there are unique bacterial microbiota signatures for each tobacco product or even brand.

In summary, our findings show that all the little cigars brands tested in this study harbor diverse bacterial communities, which differ significantly across little cigar brands and little cigar component (e.g., tobacco or wrapper). In addition, we demonstrated that storage conditions can alter the bacterial microbiota of little cigars over time. The precise public health implications of these findings remain uncertain, but it is clear that follow-up studies are needed to further characterize the tobacco bacterial communities, especially with regard to the active role they may play in altering the tobacco users’ oral microbiota, introducing potentially-harmful pathogenic bacteria into users’ oral cavities, and ultimately impacting users’ health.

## Author Contributions

AS and EM designed the study, developed the protocols, contributed to data analyses and results interpretation, and manuscript preparation. ES participated in the laboratory analyses, performed the bioinformatics and statistical analyses after sequence processing, wrote and edited the manuscript. JC participated in data analysis, interpretation of results, and manuscript editing. SC, LM, and LH performed sample processing, laboratory analyses, and contributed to data analysis and interpretation. PC contributed to study design and manuscript review. All authors read and approved the final manuscript.

## Conflict of Interest

The authors declare that the research was conducted in the absence of any commercial or financial relationships that could be construed as a potential conflict of interest.

## Supplementary Material

FIGURE S1 Scree plot showing eigenvalues for the first 100 axis.

FIGURE S2 Ordination plots of little cigar samples: **(A)** PCoA using Bray–Curtis dissimilarity of bacterial community composition: **(B)** t-SNE using Bray–Curtis dissimilarity of bacterial community composition. Colors represent little cigar brands and shapes represent little cigar portions. Ellipses are drawn at 95% confidence intervals for little cigar portion.

FIGURE S3 Ordination plot derived from PCoA using Bray–Curtis distance of bacterial community composition, separated by little cigar brand, tobacco portion, and condition. Colors signify different lots and shapes signify different days. Ellipses are drawn at 95% confidence intervals for product manufacturer.

FIGURE S4 Changes in mean relative abundance of significantly differentially abundant OTUs over time in Cheyenne Menthol Box tobacco. Colors indicate the storage conditions.

FIGURE S5 Changes in mean relative abundance of significantly differentially abundant OTUs over time in Cheyenne Menthol Box wrapper. Colors indicate the storage conditions.

FIGURE S6 Average relative abundance of biomarker OTUs in little cigar components.

TABLE S1 List of study samples with associated meta and sequencing data.
