# Supplementary material for: The Bacterial Communities of Little Cigars and Cigarillos Are Dynamic Over Time and Varying Storage Conditions
Source: Front Microbiol. 2019 Oct 25;10:2371. doi: 10.3389/fmicb.2019.02371 (PMC6824217; doi:10.3389/fmicb.2019.02371)
Supplement: FIGURE S1 — Scree plot showing eigenvalues for the first 100 axis. [file Data_Sheet_1.PDF]

Supplementary Figure 1

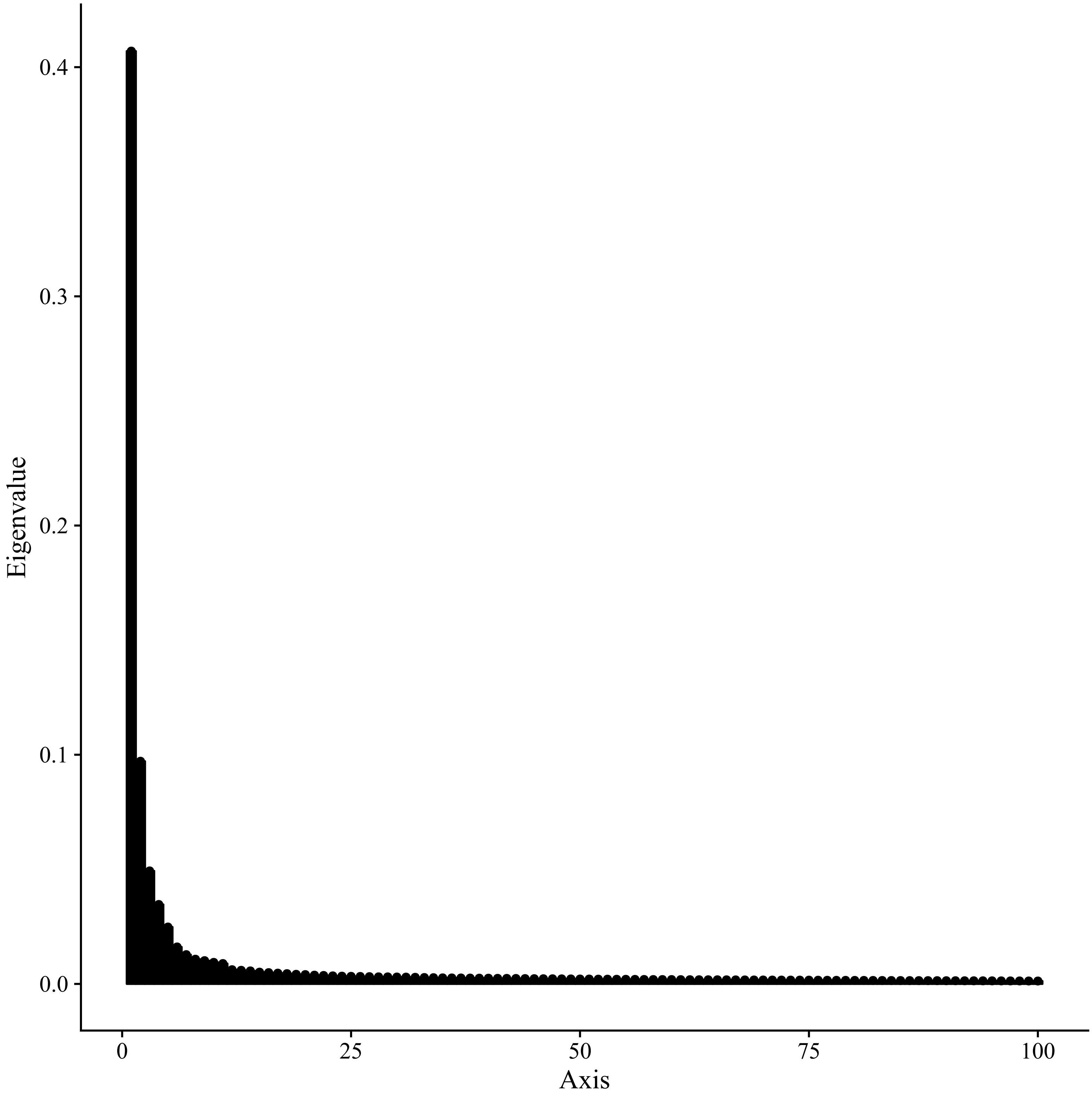

Supplementary Figure 2

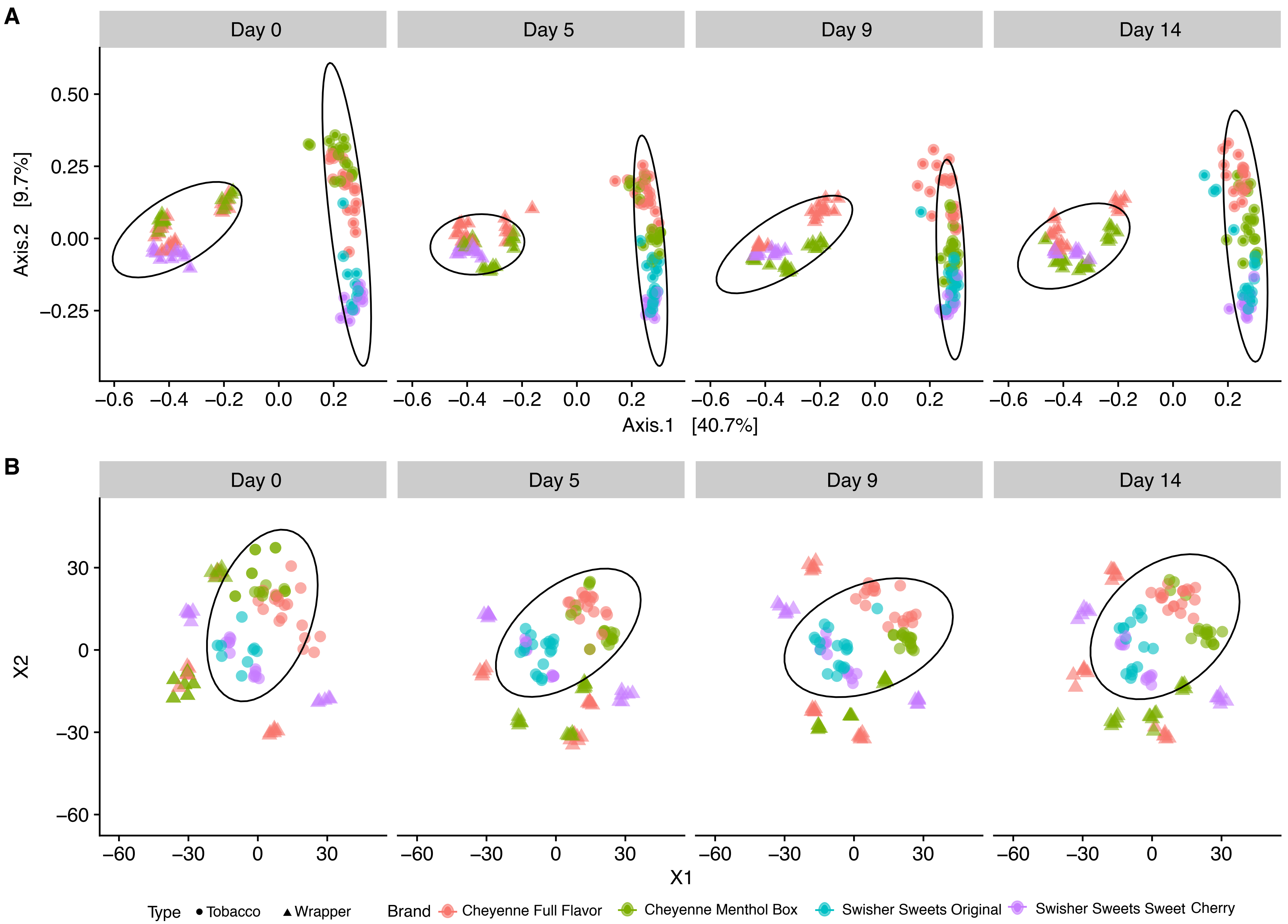

Supplementary Figure 3

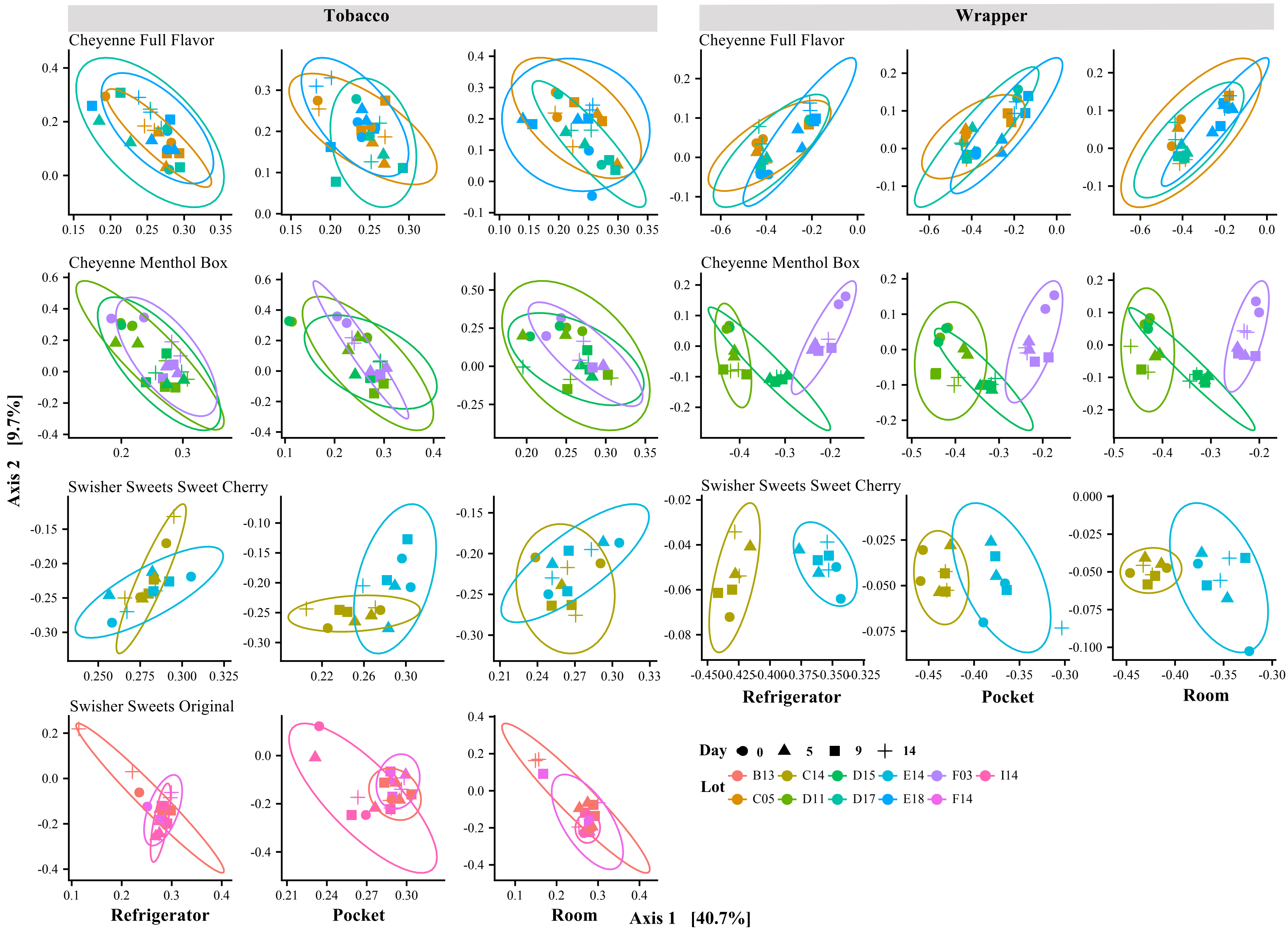

Supplementary Figure 4

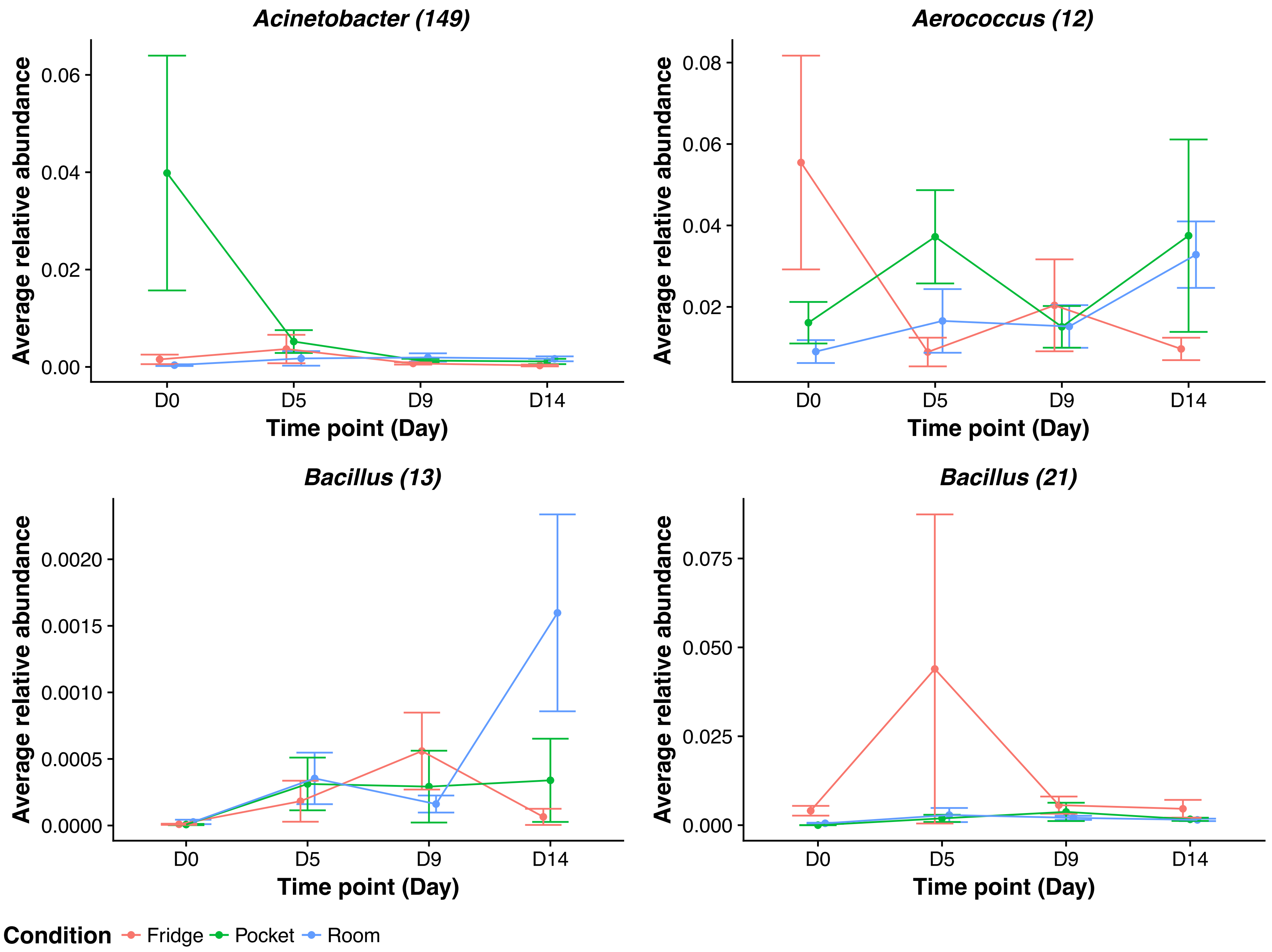

Supplementary Figure 4: Changes in mean relative abundance of significantly differentially abundant OTUs over time in Cheyenne Menthol Box tobacco component.

Supplementary Figure 4 (Cont'd)

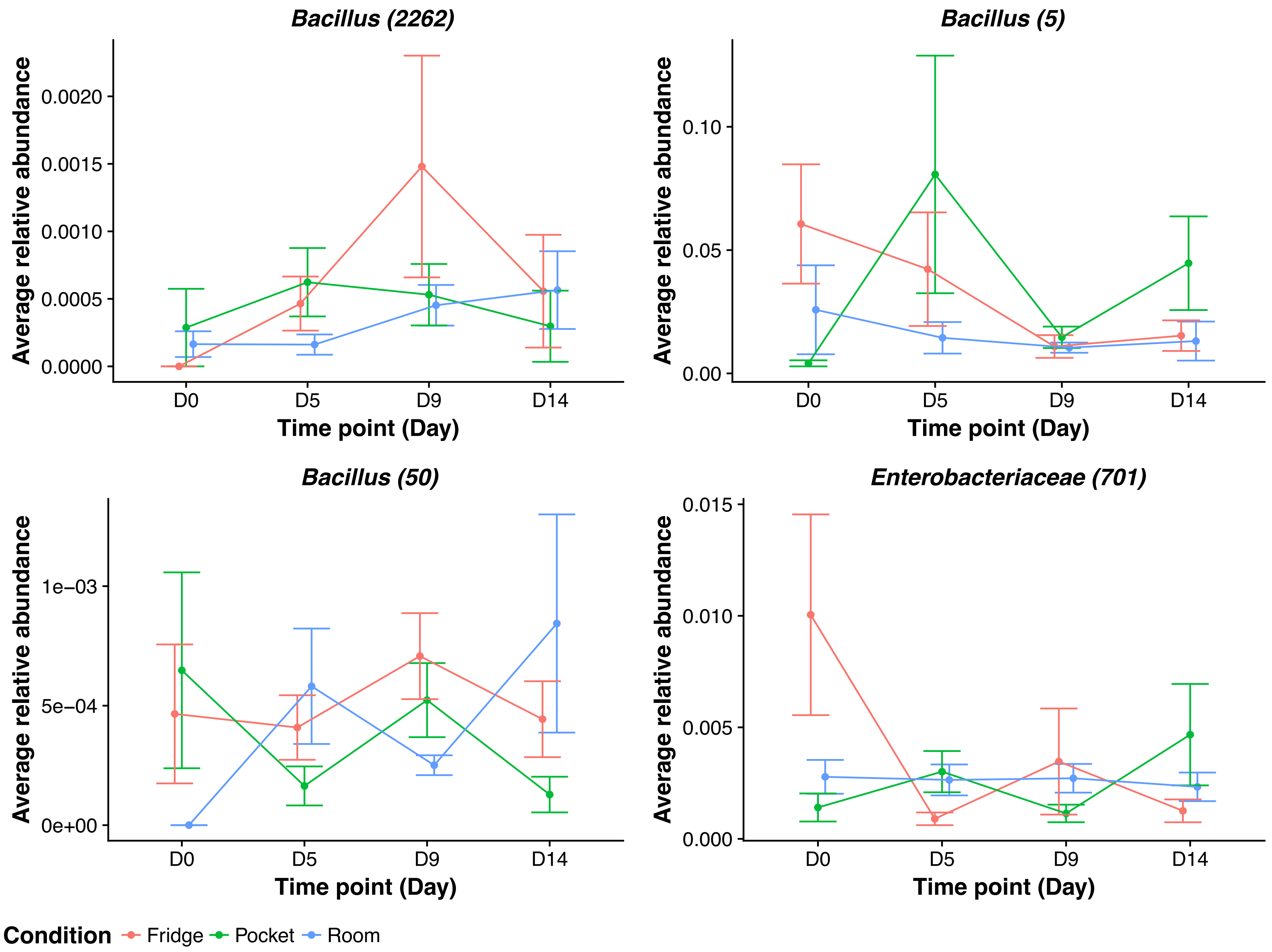

Supplementary Figure 4: Changes in mean relative abundance of significantly differentially abundant OTUs over time in Cheyenne Menthol Box tobacco component.

Supplementary Figure 4 (Cont'd)

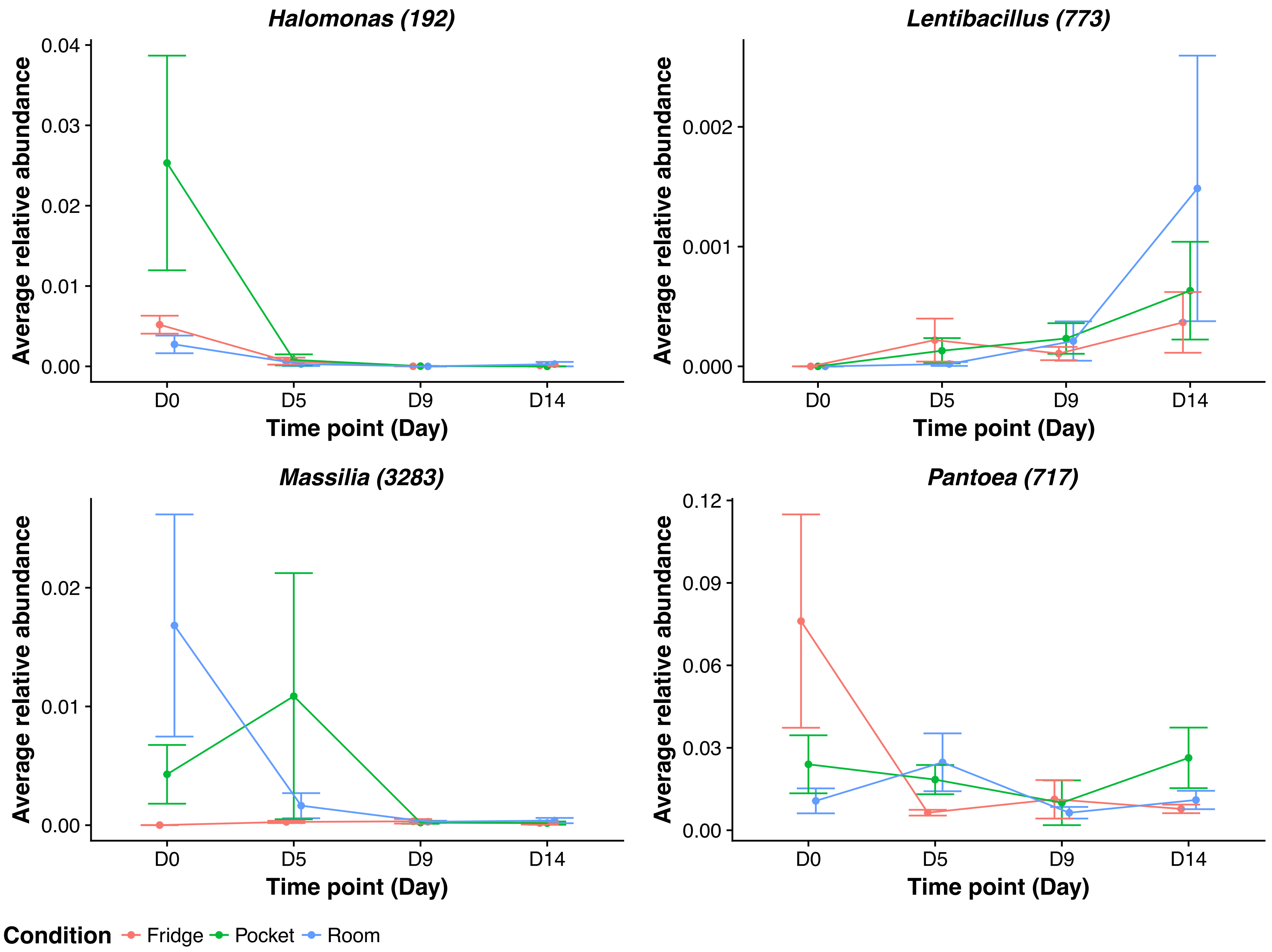

Supplementary Figure 4: Changes in mean relative abundance of significantly differentially abundant OTUs over time in Cheyenne Menthol Box tobacco component.

Supplementary Figure 4 (Cont'd)

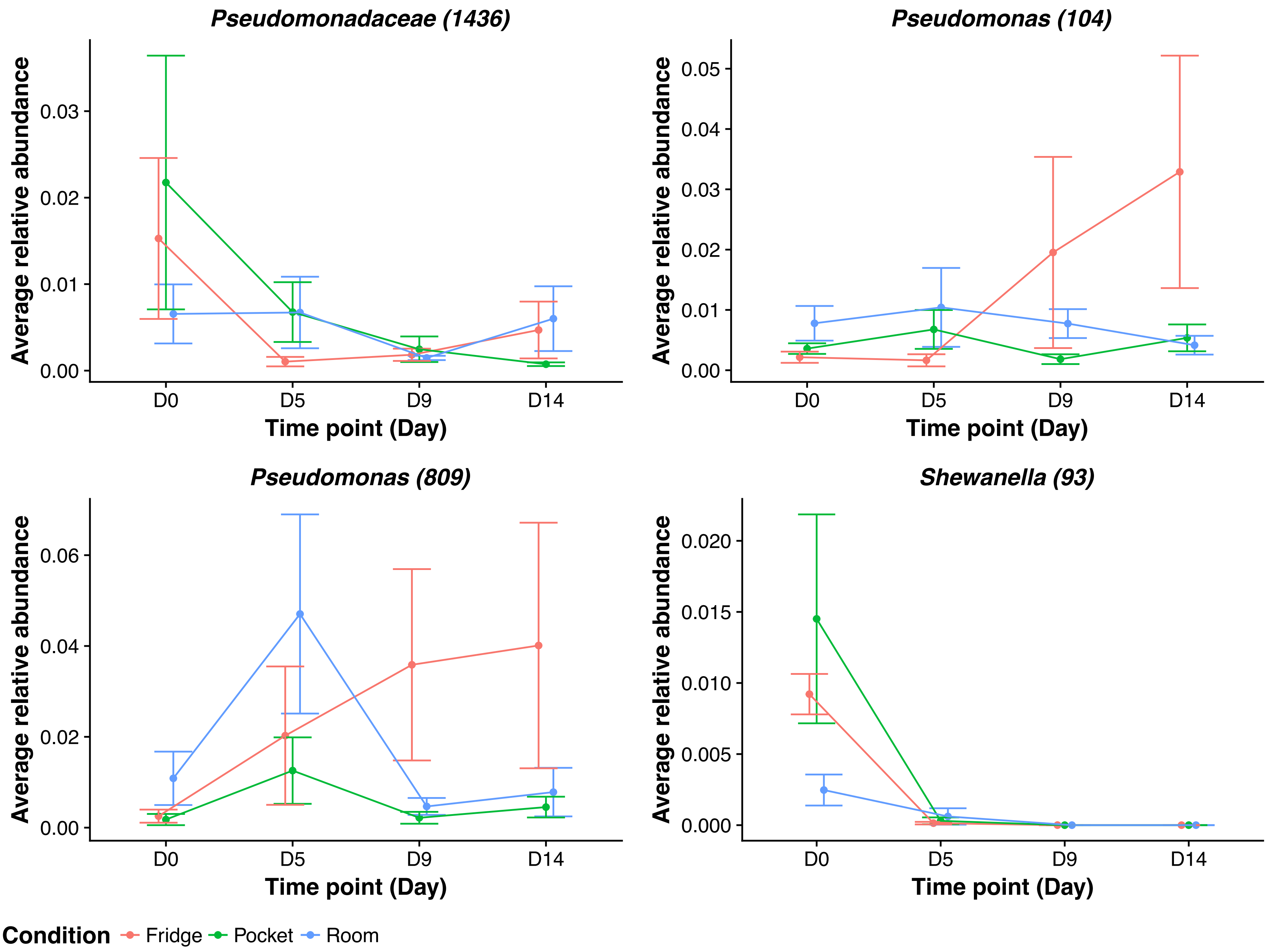

Supplementary Figure 4: Changes in mean relative abundance of significantly differentially abundant OTUs over time in Cheyenne Menthol Box tobacco component.

Supplementary Figure 4 (Cont'd)

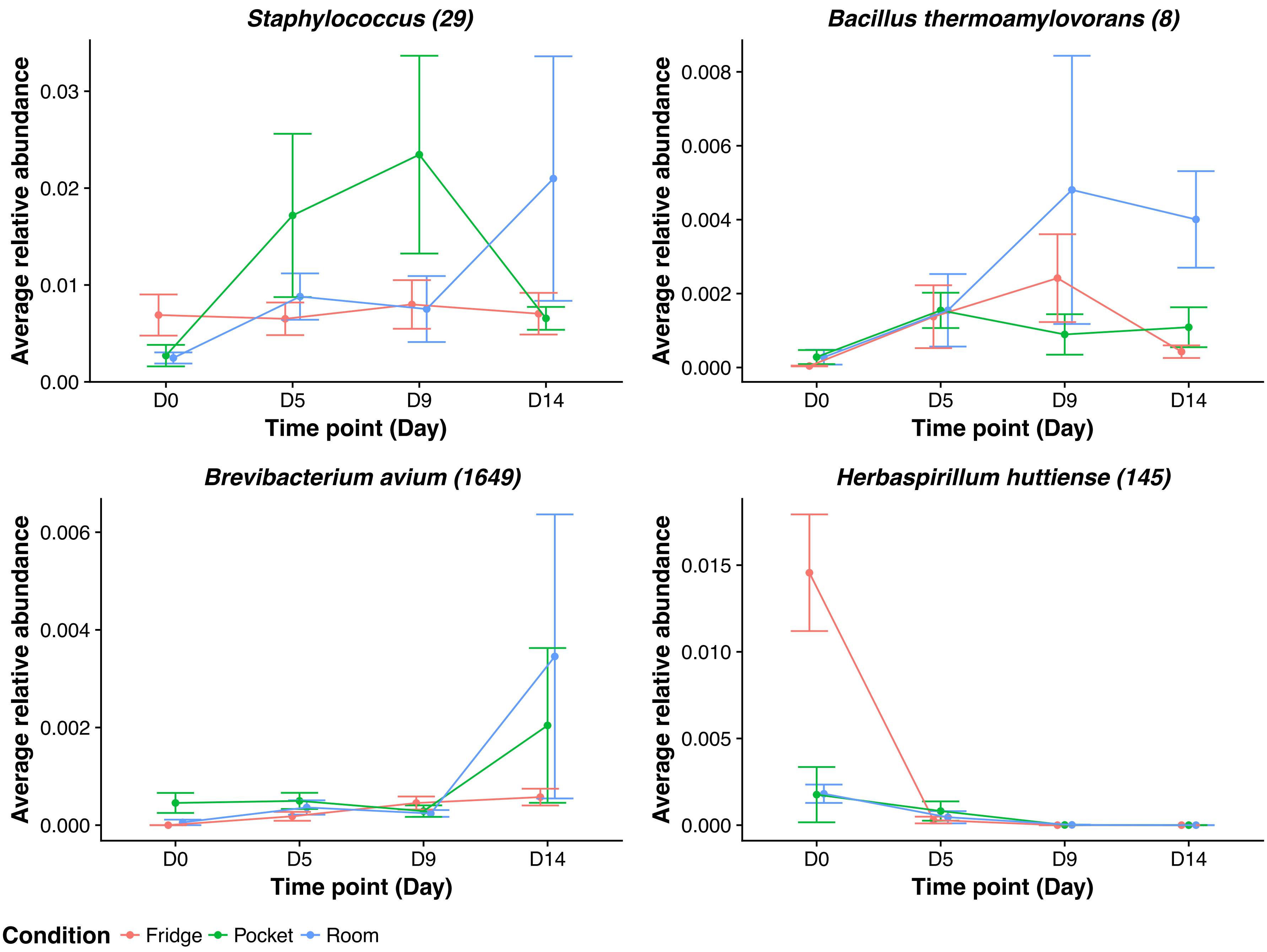

Supplementary Figure 4: Changes in mean relative abundance of significantly differentially abundant OTUs over time in Cheyenne Menthol Box tobacco component.

Supplementary Figure 4 (Cont'd)

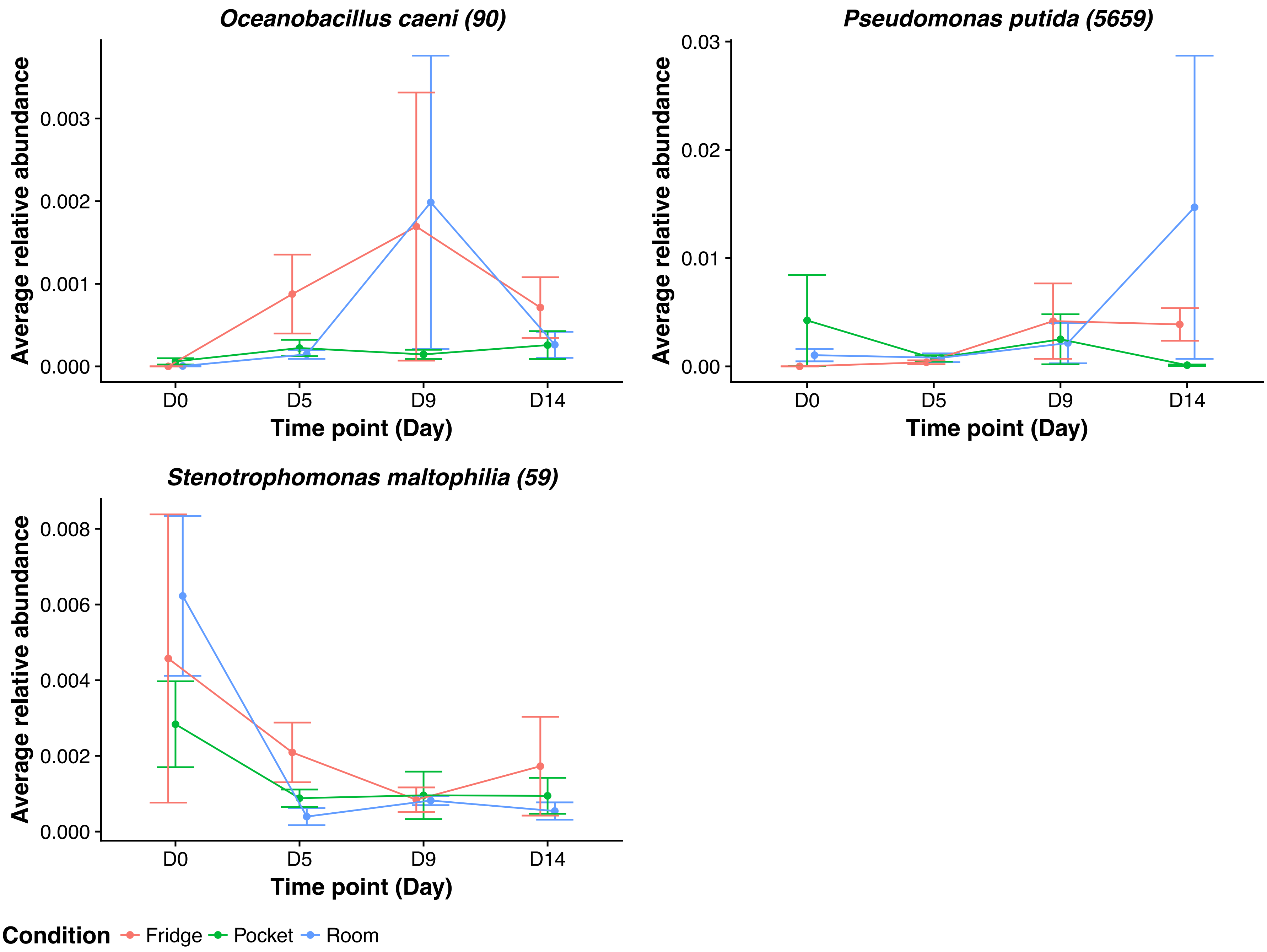

Supplementary Figure 4: Changes in mean relative abundance of significantly differentially abundant OTUs over time in Cheyenne Menthol Box tobacco component.

Supplementary Figure 5

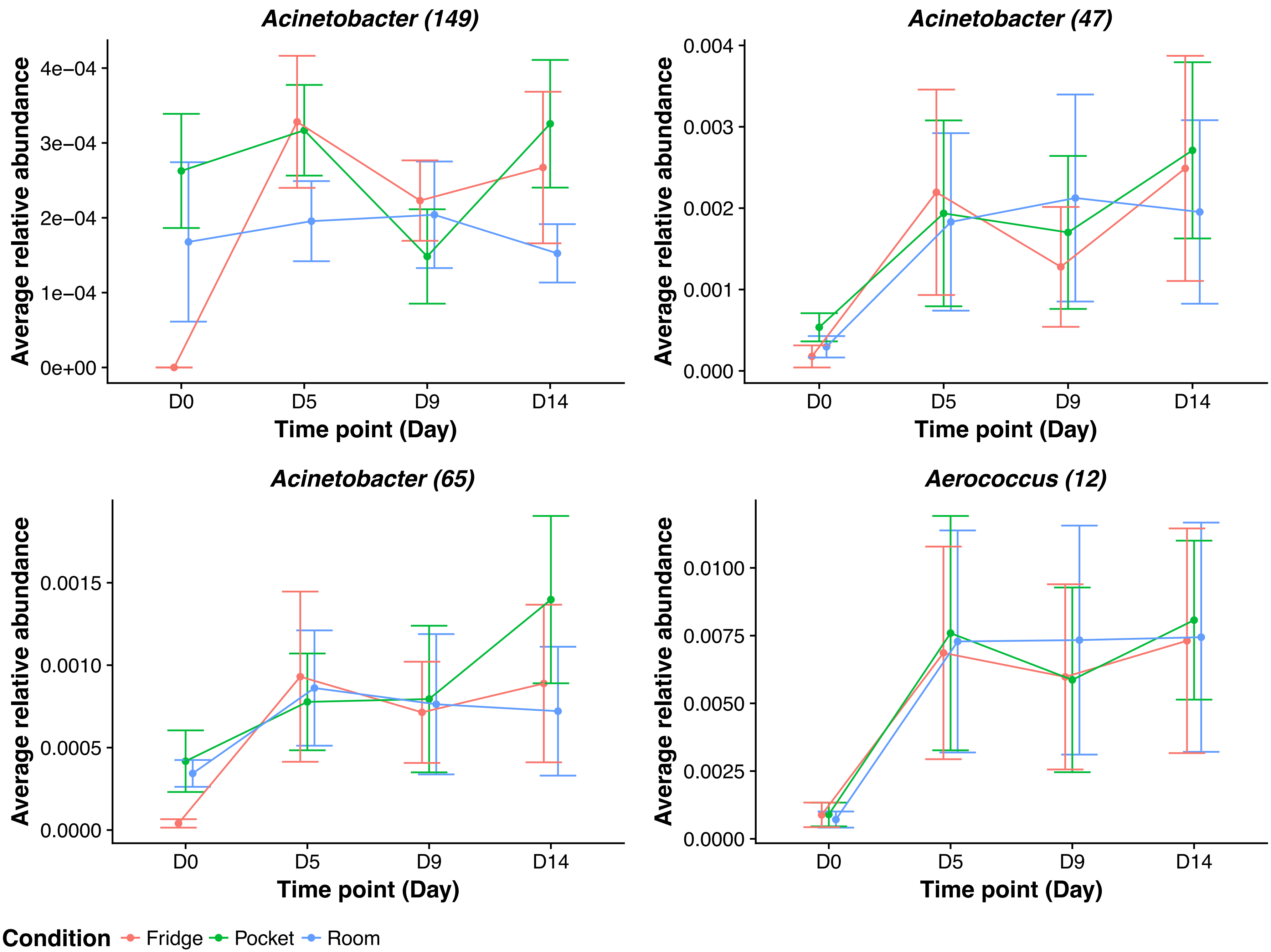

Supplementary Figure 5: Changes in mean relative abundance of significantly differentially abundant OTUs over time in Cheyenne Menthol Box wrapper component

Supplementary Figure 5 (Cont'd)

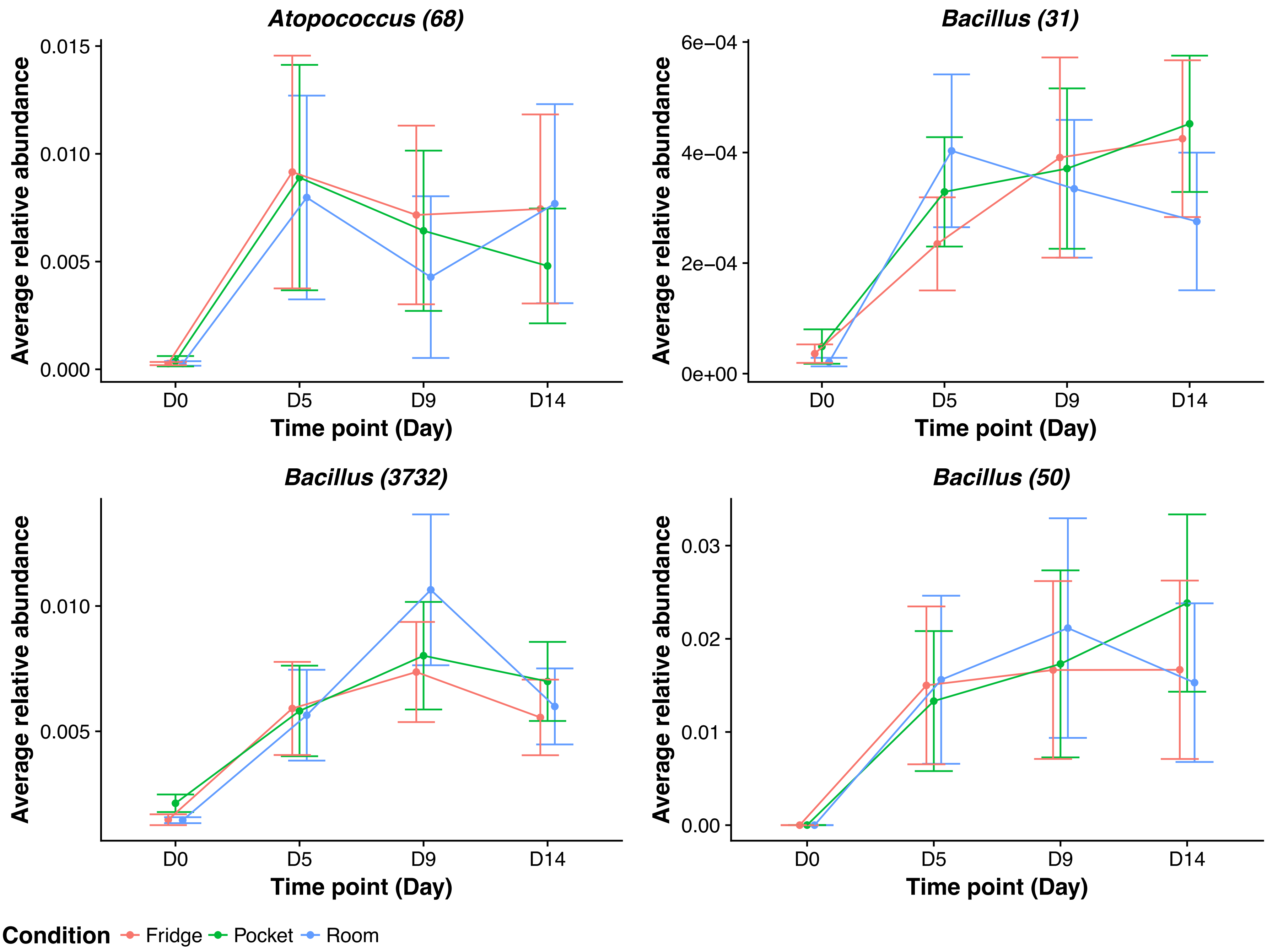

Supplementary Figure 5: Changes in mean relative abundance of significantly differentially abundant OTUs over time in Cheyenne Menthol Box wrapper component

Supplementary Figure 5 (Cont'd)

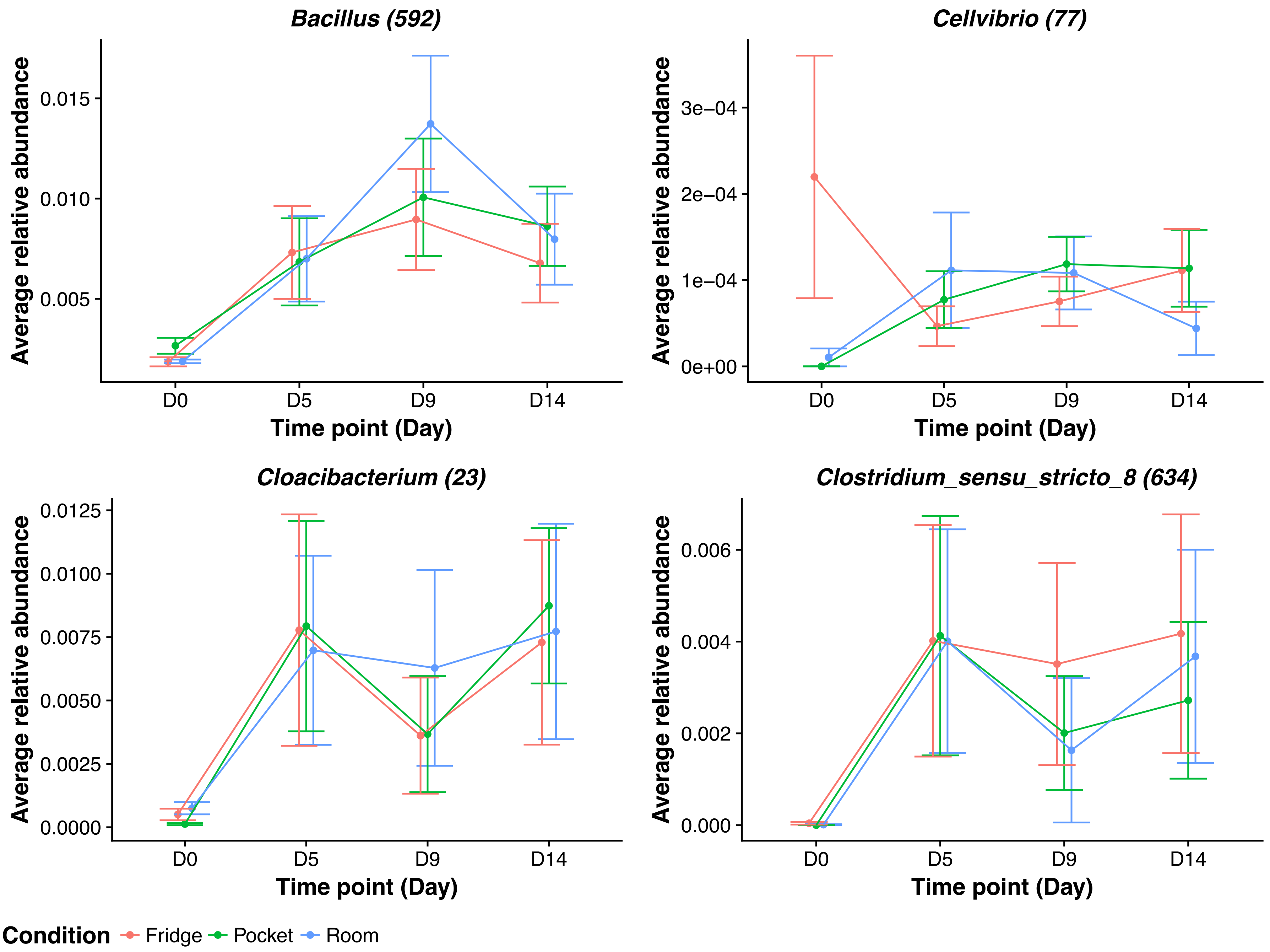

Supplementary Figure 5: Changes in mean relative abundance of significantly differentially abundant OTUs over time in Cheyenne Menthol Box wrapper component

Supplementary Figure 5 (Cont'd)

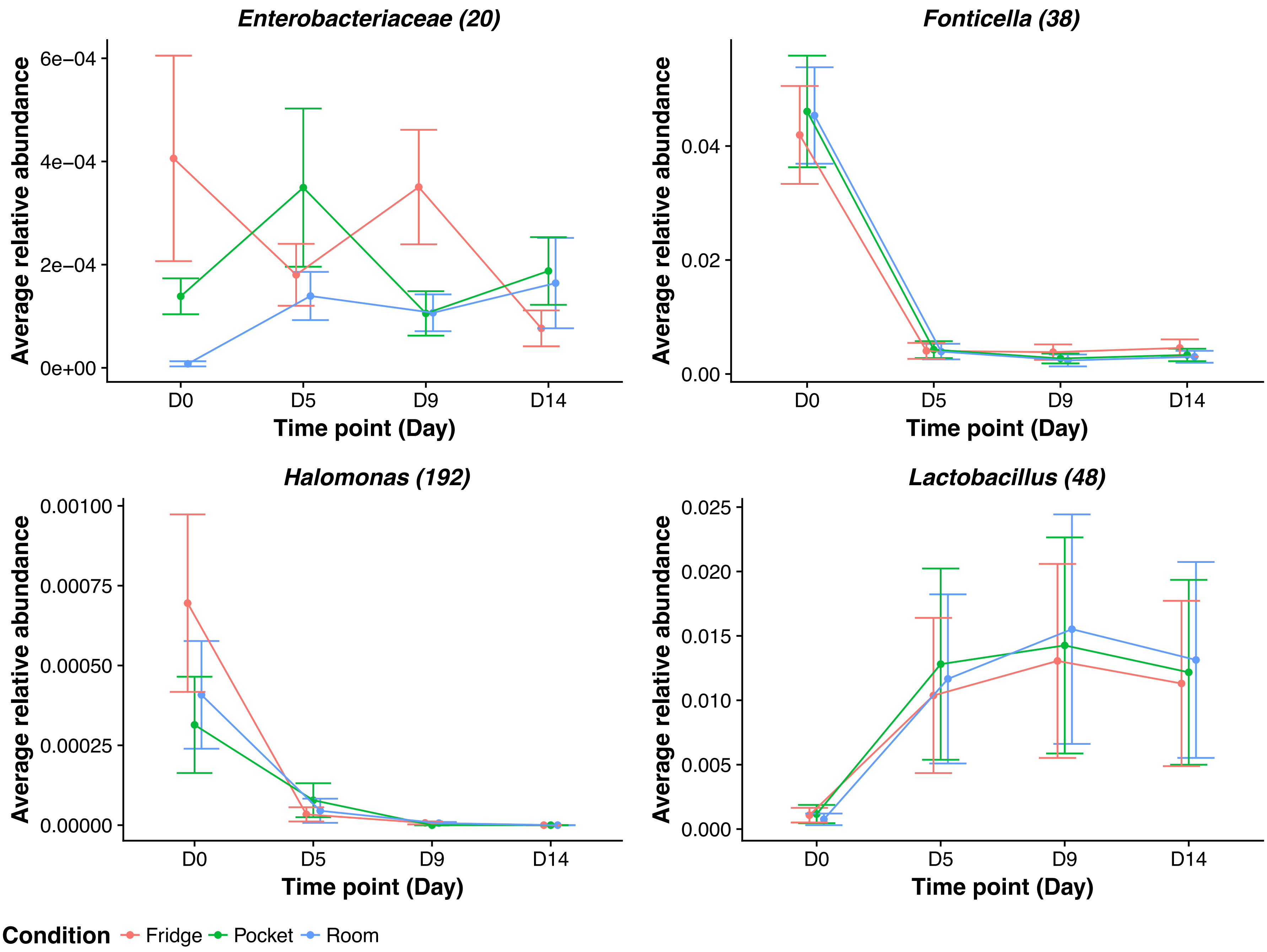

Supplementary Figure 5: Changes in mean relative abundance of significantly differentially abundant OTUs over time in Cheyenne Menthol Box wrapper component

Supplementary Figure 5 (Cont'd)

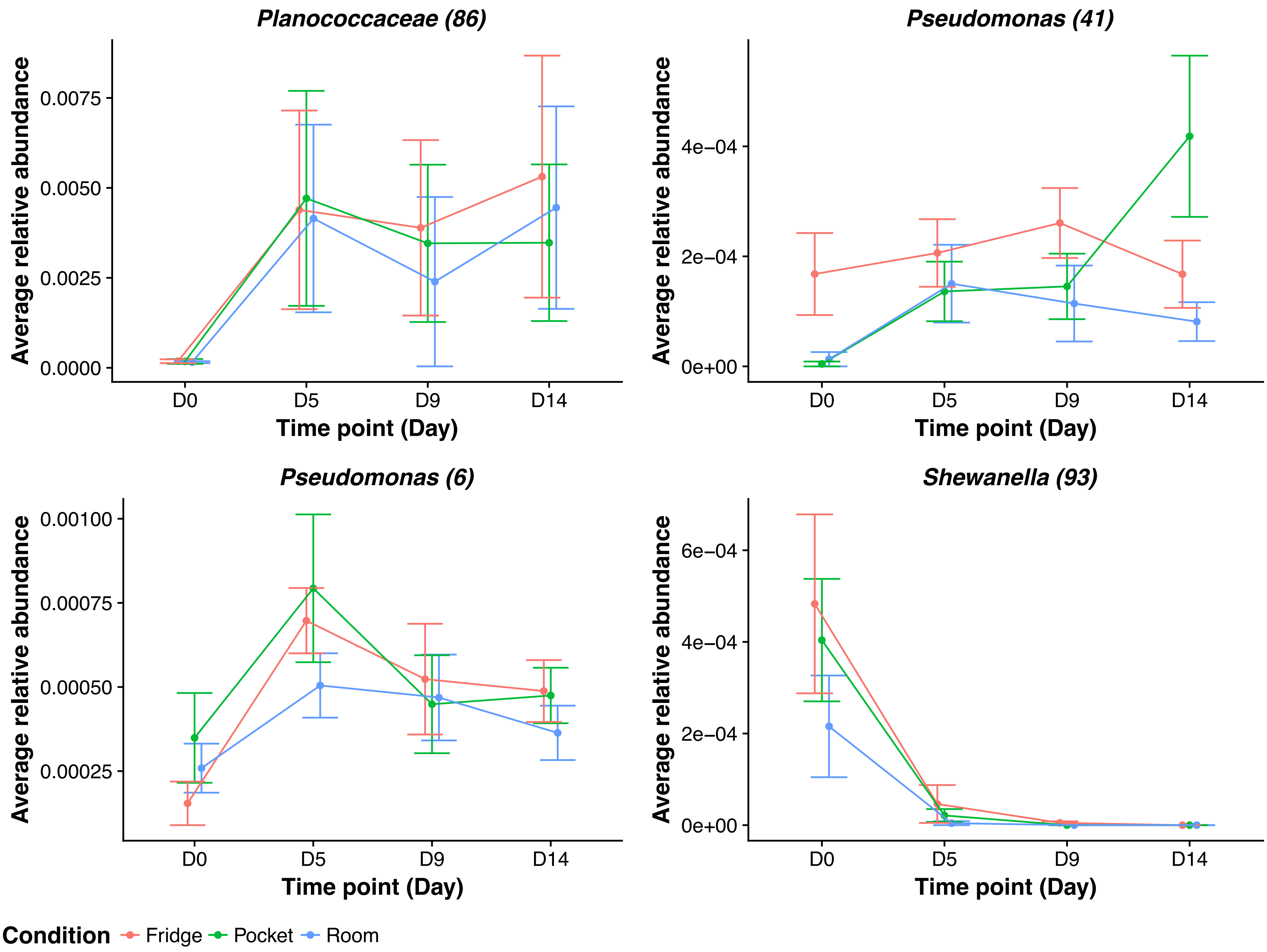

Supplementary Figure 5: Changes in mean relative abundance of significantly differentially abundant OTUs over time in Cheyenne Menthol Box wrapper component

Supplementary Figure 5 (Cont'd)

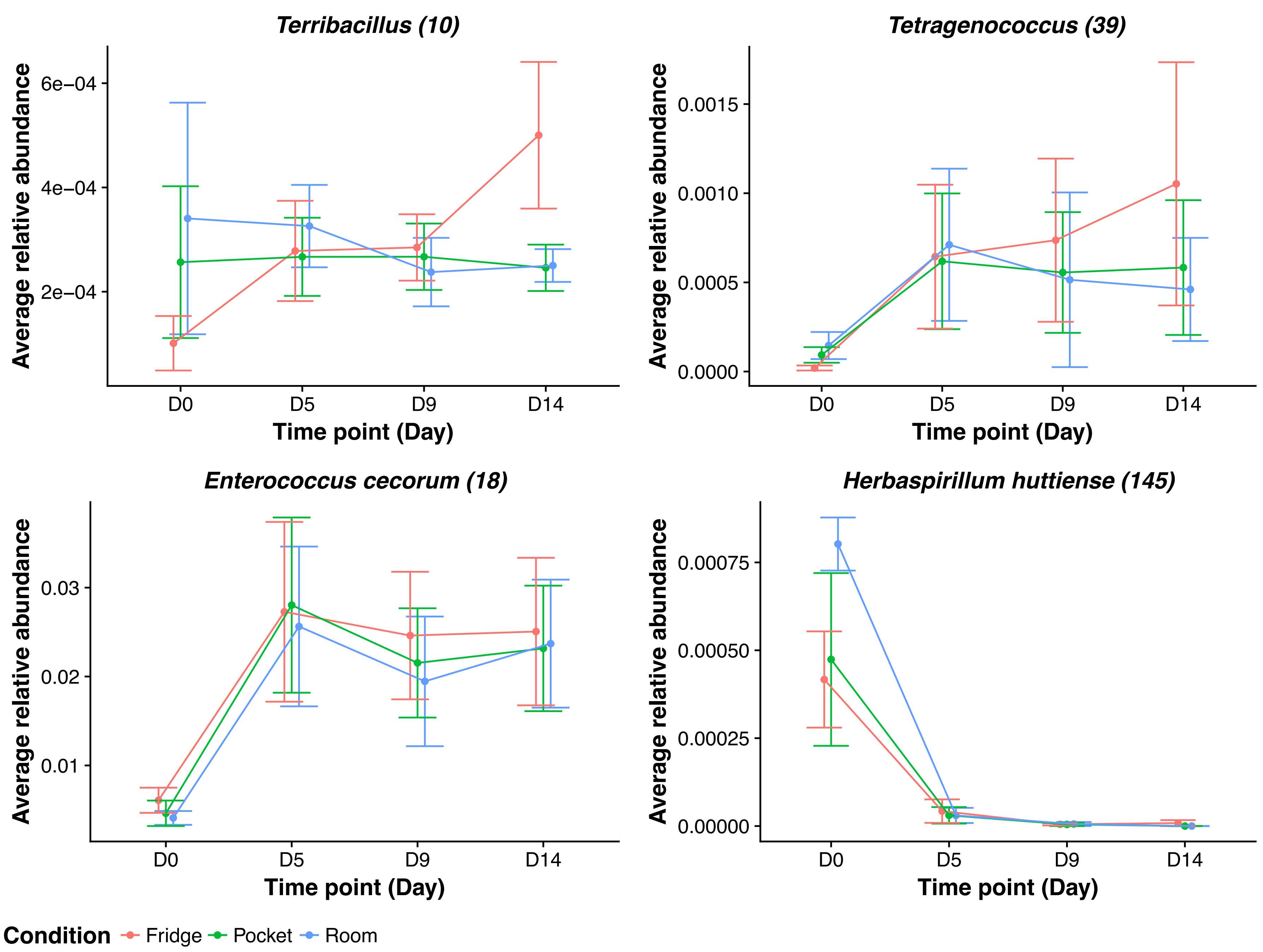

Supplementary Figure 5: Changes in mean relative abundance of significantly differentially abundant OTUs over time in Cheyenne Menthol Box wrapper component

Supplementary Figure 5 (Cont'd)

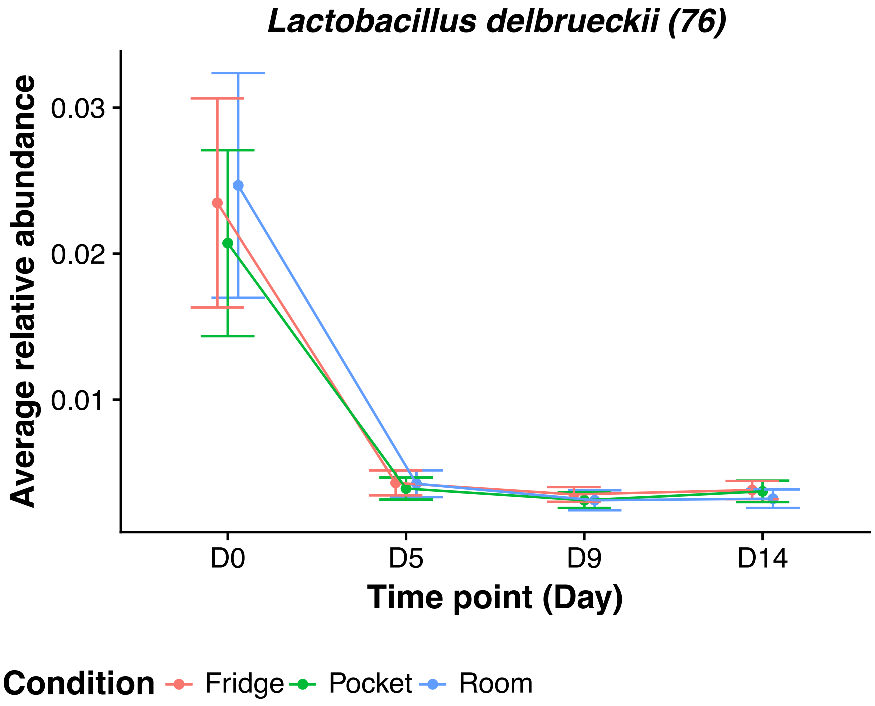

Supplementary Figure 5: Changes in mean relative abundance of significantly differentially abundant OTUs over time in Cheyenne Menthol Box wrapper component

Supplementary Figure 6

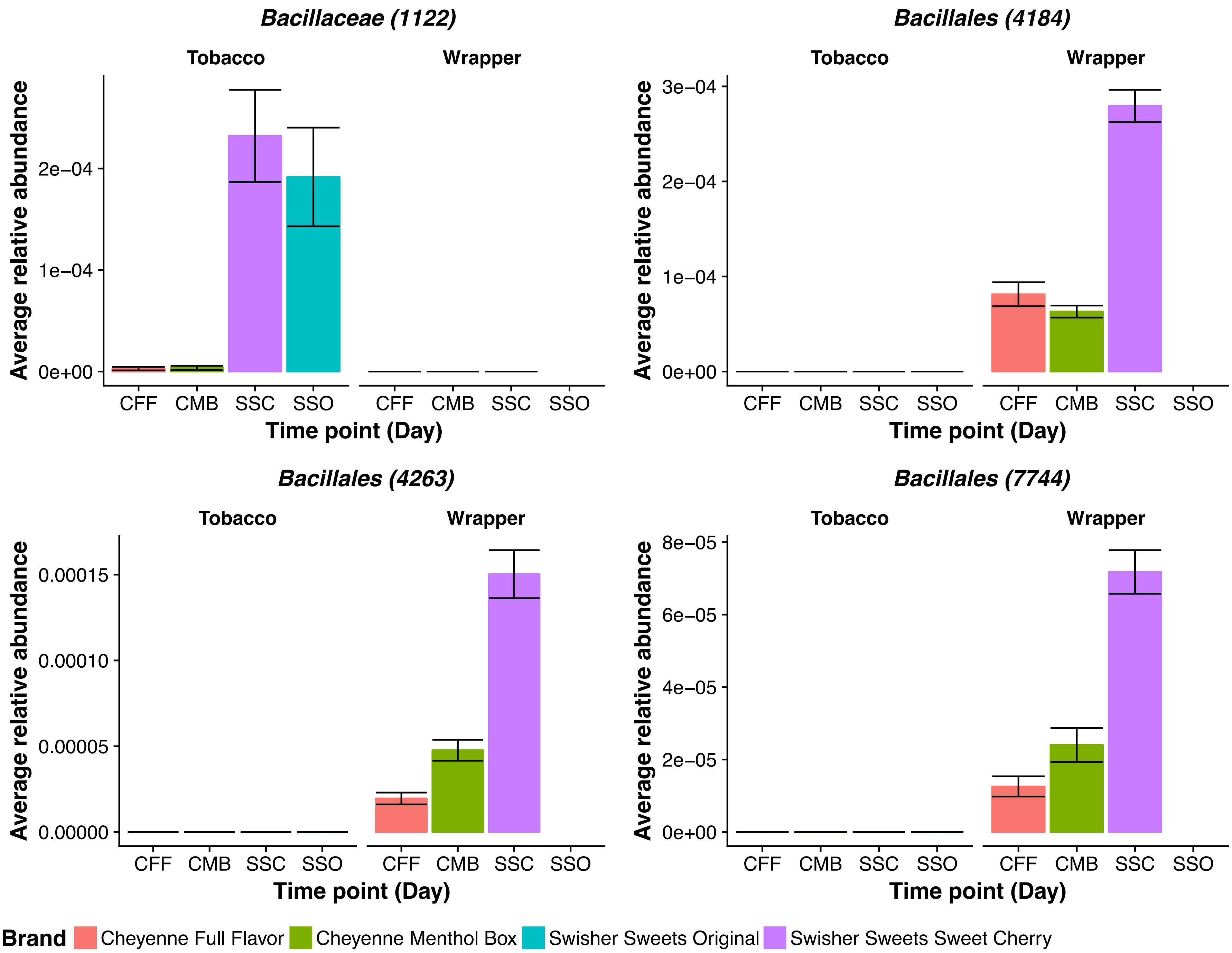

Supplementary Figure 6: Average relative abundance of biomarker OTUs in little cigar components.

Supplementary Figure 6 (Cont'd)

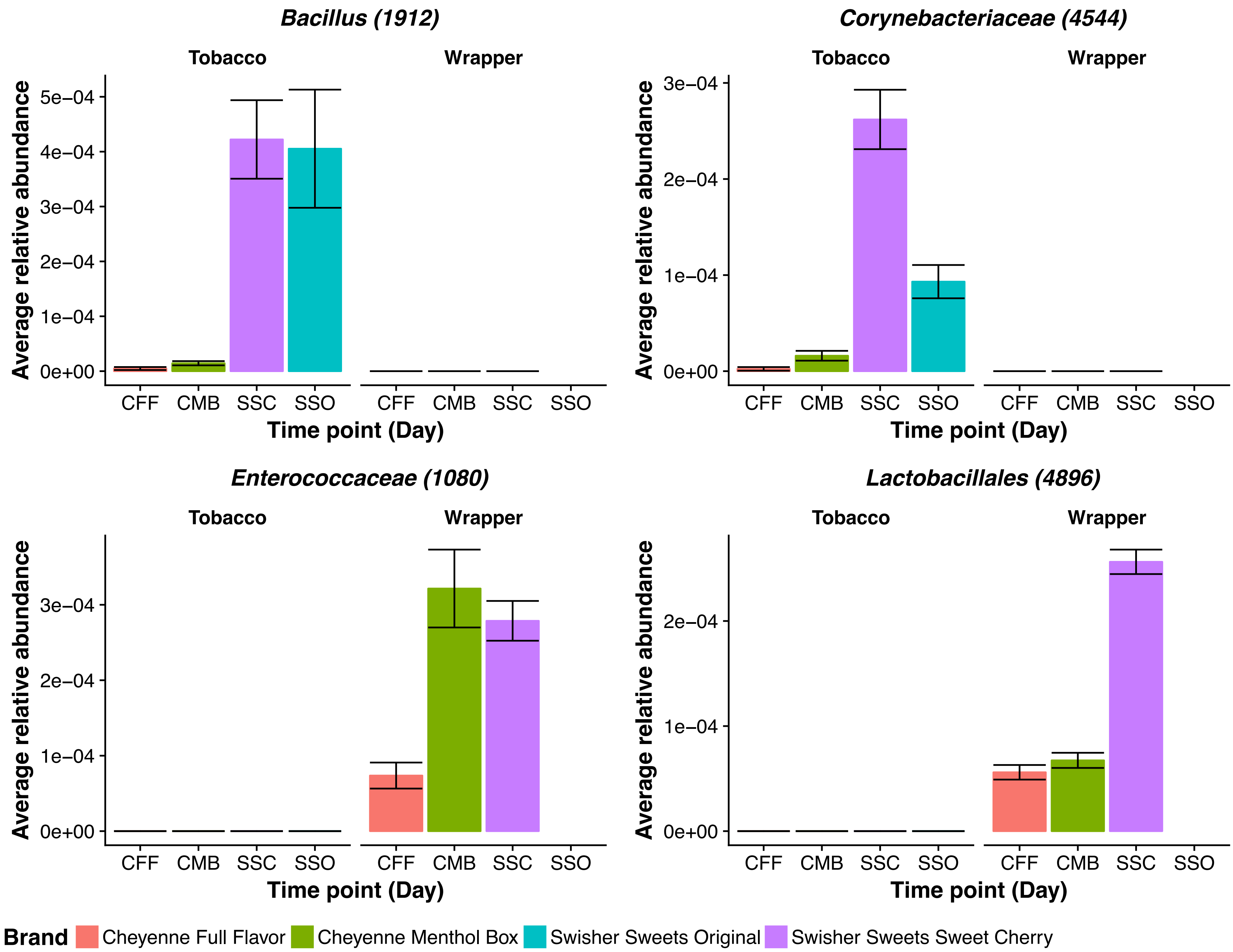

Supplementary Figure 6: Average relative abundance of biomarker OTUs in little cigar components.

Supplementary Figure 6 (Cont'd)

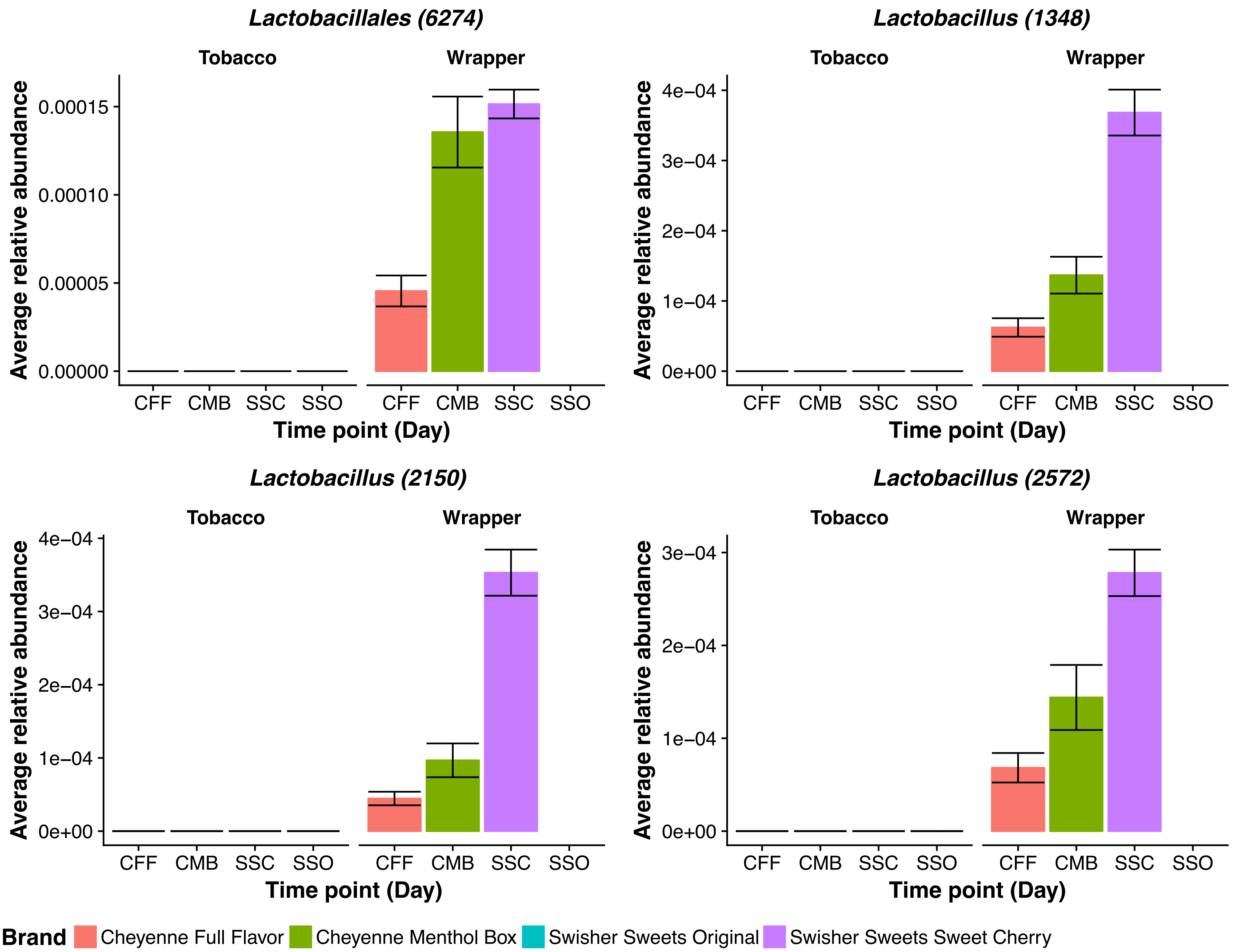

Supplementary Figure 6: Average relative abundance of biomarker OTUs in little cigar components.

Supplementary Figure 6 (Cont'd)

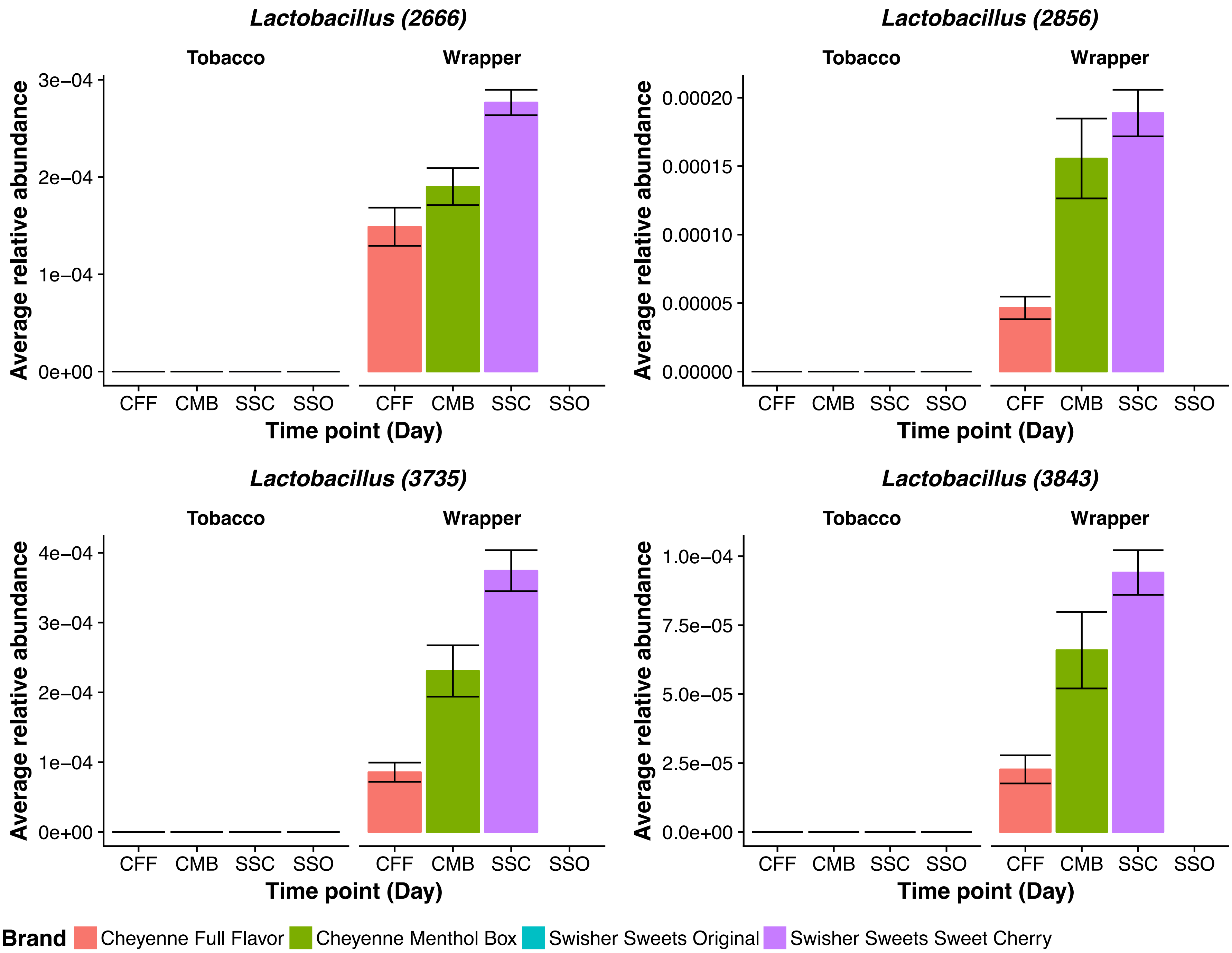

Supplementary Figure 6: Average relative abundance of biomarker OTUs in little cigar components.

Supplementary Figure 6 (Cont'd)

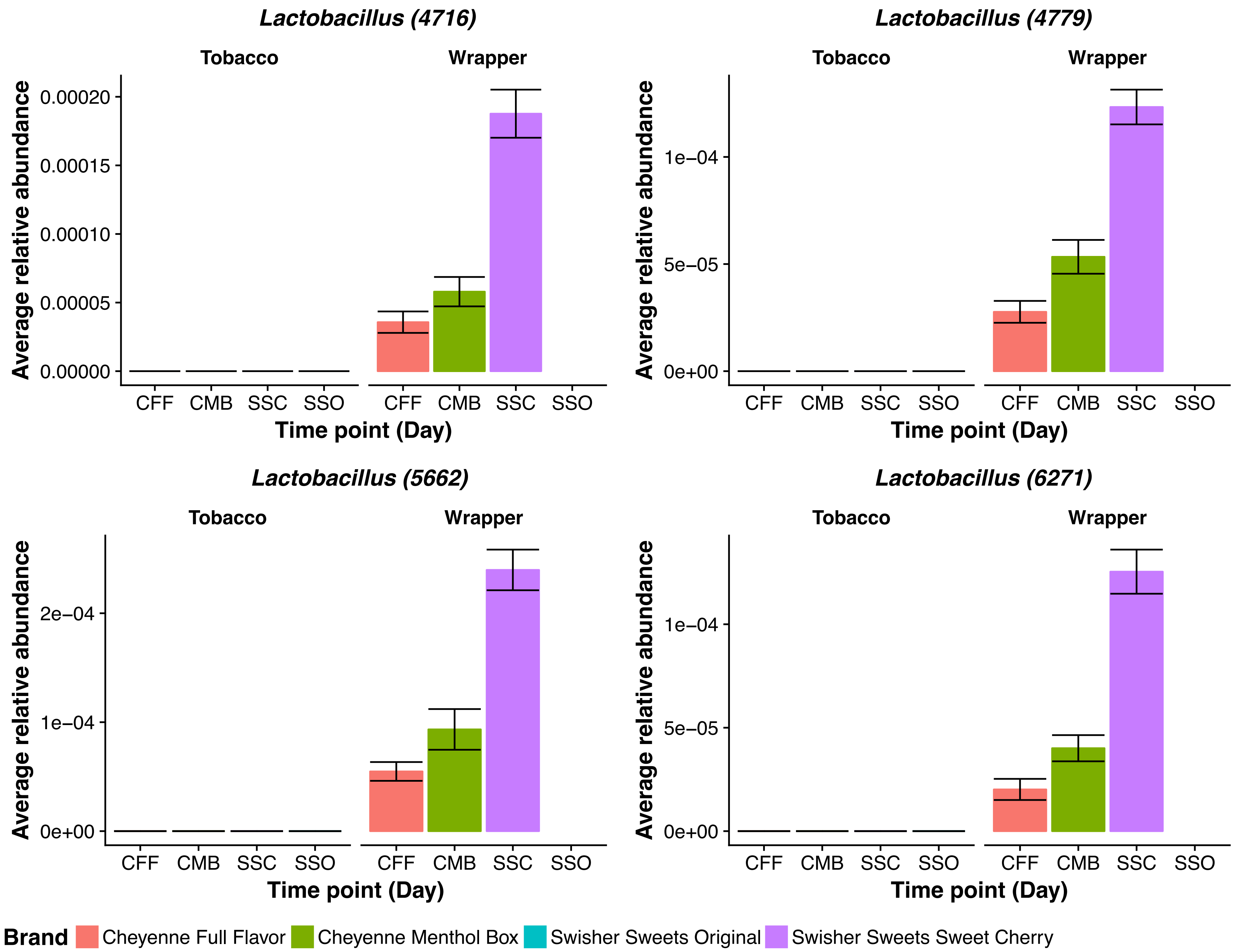

Supplementary Figure 6: Average relative abundance of biomarker OTUs in little cigar components.

Supplementary Figure 6 (Cont'd)

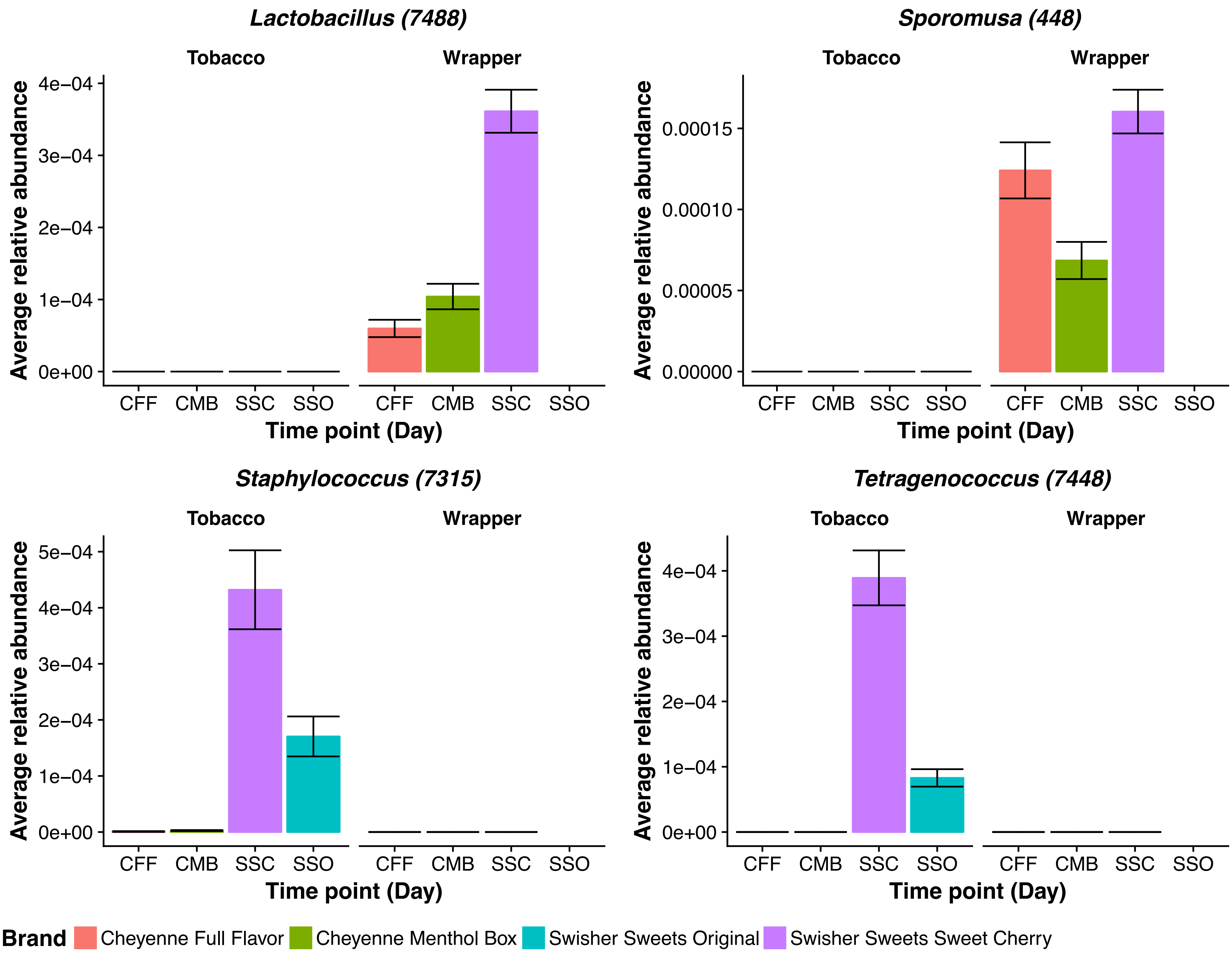

Supplementary Figure 6: Average relative abundance of biomarker OTUs in little cigar components.
